# Tuning metal ion affinity in acyclic phenanthrene schiff bases: comparative study of ethylene and phenylene linkers

**DOI:** 10.1039/d5ra03617h

**Published:** 2025-08-11

**Authors:** Haritha C, Swathi M, Chinna Ayya Swamy P

**Affiliations:** a Main Group Organometallics Optoelectronic Materials and Catalysis Lab, Department of Chemistry, National Institute of Technology Calicut-673601 India swamy@nitc.ac.in

## Abstract

We have successfully designed and synthesized two structurally simple salen-type Schiff base probes, designated as SB-1 and SB-2, for the selective detection of biologically and environmentally relevant metal ions. Fluorescence studies revealed that SB-1 exhibits a distinct “turn-on” fluorescence response in the presence of Zn^2+^, Mg^2+^, Na^+^, and K^+^ ions, while SB-2 demonstrated a selective fluorescence enhancement exclusively for Zn^2+^ ions. In addition to its fluorescence response, SB-1 displayed distinct colorimetric changes upon interaction with Zn^2+^, Cu^2+^, Mg^2+^, Na^+^, and K^+^ ions, highlighting its broad-spectrum sensing capability. In contrast, SB-2 exhibited selective colorimetric responses only toward Zn^2+^ and Cu^2+^ ions. These results underscore the dual-mode sensing potential of the probes, with SB-1 offering broader ion recognition and SB-2 demonstrating higher selectivity. To gain insights into the interaction mechanism and validate the spectral changes observed experimentally, density functional theory (DFT) calculations were performed. The computational results supported the experimental findings, confirming significant electronic transitions associated with metal ion binding and providing a detailed understanding of the coordination environment and binding modes of SB-1 and SB-2 with various metal ions. ^1^H NMR titration studies further substantiated these results by revealing that the metal ions coordinate with the imine (C=N) moiety at the core of the Schiff base structure, consistent with the UV-visible absorption and fluorescence spectroscopy data. The binding stoichiometry between the probes and metal ions (Zn^2+^, Cu^2+^, Mg^2+^, Na^+^, and K^+^) was determined to be 1 : 1, as confirmed through Job's plot analysis and supported by UV-Vis, fluorescence, DFT, and ^1^H NMR (for Zn^2+^) studies. The binding affinities were quantified using the Benesi–Hildebrand method, with association constants (*K*_a_) found to be in the range of 0.88–2.28 × 10^3^ M^−1^. The limit of detection (LOD) for each metal ion was calculated to be in the micromolar (μM) range (0.1 to 0.05 μM), demonstrating the high sensitivity of the probes for practical sensing applications.

## Abstract 2

We have successfully designed and synthesized two structurally simple salen-type Schiff base probes, designated as SB-1 and SB-2, for the selective detection of biologically and environmentally relevant metal ions.

## Graphical abstract

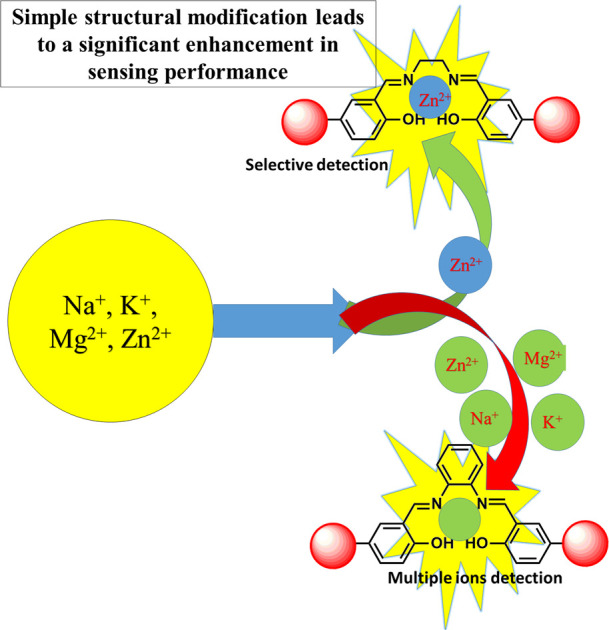

## 1. Introduction

Metal ion sensing is a critical area of research with far-reaching implications in both biological and environmental sciences.^1^ Within this broad field, multi-metal ion sensing is especially valuable, as biological systems are inherently complex and often involve the simultaneous presence and interaction of multiple metal ions.^2^ These ions play vital roles in cellular processes such as enzymatic catalysis, ion transport, gene regulation, and signal transduction.^3^ Accurately detecting and distinguishing between these ions, particularly in dynamic or physiologically relevant environments, remains a significant challenge due to their similar charge-to-radius ratios and overlapping coordination preferences.^4^ Among the biologically essential metal ions, zinc (Zn^2+^) occupies a central position due to its abundance and multifunctionality in the human body. It is involved in DNA synthesis, transcriptional regulation, immune function, and neurotransmission, functioning either as a catalytic or structural cofactor.^5^ Unlike redox-active metals like iron or copper, Zn^2+^ is redox-inert but exhibits a wide range of coordination geometries and ligand affinities, making it an ideal target for selective fluorescence sensing.^6^ Dysregulation of zinc homeostasis has been implicated in numerous pathological conditions, including Alzheimer's disease, epilepsy, diabetes, and prostate cancer.^7^ Environmentally, zinc contamination can disrupt microbial ecology and plant growth, underscoring the importance of its detection in soil and water samples.^8^ Other metal ions such as sodium (Na^+^), potassium (K^+^), magnesium (Mg^2+^), and copper (Cu^2+^) are also indispensable for proper physiological functioning. Na^+^ and K^+^ play key roles in maintaining osmotic balance, nerve impulse transmission, and muscle contraction.^9^ Mg^2+^ acts as a cofactor in over several enzymatic reactions, including those involved in ATP metabolism and nucleic acid synthesis.^10^ Cu^2+^, while essential in trace amounts for redox enzymes and iron metabolism, can be highly toxic when accumulated in excess, contributing to oxidative stress and neurodegenerative diseases.^11^ The environmental and physiological significance of these ions, coupled with the narrow window between their beneficial and toxic levels, highlights the urgent need for accurate and efficient detection methods.^12^

To address this need, traditionally metal ion detection has relied on techniques such as atomic absorption spectroscopy (AAS) and inductively coupled plasma mass spectrometry (ICP-MS), which offer high sensitivity but require expensive instrumentation, extensive sample preparation, and are not amenable to real-time or *in situ* analysis.^13^ In contrast, fluorescence-based sensors have emerged as powerful alternatives owing to their high sensitivity, operational simplicity, cost-effectiveness, and compatibility with live-cell imaging and real-time monitoring.^14^ A wide variety of photophysical mechanisms – such as chelation-enhanced fluorescence (CHEF), photoinduced electron transfer (PET), internal charge transfer (ICT), fluorescence resonance energy transfer (FRET), and excimer/exciplex formation – have been employed in sensor design.^15^ Among these, PET-based sensors are particularly attractive for their “*turn-on*” response mechanism, in which fluorescence is quenched in the free state and restored upon metal ion coordination.^16,20^ This mechanism ensures high signal-to-noise ratios and makes PET sensors especially useful for detecting trace levels of metal ions.^17^ In recent years, phenanthrene has gained attention as a promising but underutilized fluorophore in sensor development.^18^ Its rigid fused-ring structure, high fluorescence quantum yield, and excellent photostability make it ideal for robust sensing applications. Despite these advantages, phenanthrene-based systems have been less explored compared to more commonly used fluorophores like coumarin, anthracene, or triphenylamine.^16*c*^

In the present study, we report the development of two novel Schiff base fluorescent probes SB-1 and SB-2 anchored on a phenanthrene core. These ligands were synthesized *via* simple and high-yielding condensation reactions involving 5-(4*a*,4*b*-dihydrophenanthren-9-yl)-2-hydroxybenzaldehyde and either benzene-1,2-diamine (SB-1) or ethane-1,2-diamine (SB-2). The photophysical behavior and metal ion sensing properties of both receptors were extensively evaluated using UV-Vis absorption and fluorescence spectroscopy in DMSO. One of the most remarkable and unexpected findings of this work is the multi-metal ion sensing capability of SB-1, which exhibits fluorescence enhancement in the presence of a suite of biologically relevant metal ions, including Na^+^, K^+^, Mg^2+^, Cu^2+^, and Zn^2+^. This broad yet selective responsiveness is particularly noteworthy given that the detection of monovalent ions such as Na^+^ and K^+^, and even divalent Mg^2+^ and Zn^2+^, through fluorescence “*turn-on*” sensors is rarely reported in the literature.^3,8^ These ions typically exhibit weak binding affinities due to their high hydration energies and lack of strong coordination tendencies, making them challenging targets for optical sensing.^3,9^ The ability of SB-1 to detect these metal ions suggests a finely tuned binding cavity and a favorable spatial arrangement around the phenanthrene-based Schiff base, possibly facilitating interactions that enhance fluorescence upon ion binding. In contrast, SB-2 demonstrates high selectivity, exhibiting a significant fluorescence *turn-on* response exclusively in the presence of Zn^2+^, making it an excellent candidate for selective zinc sensing in complex biological environments where other metal ions may be present. This study not only underscores the potential of phenanthrene as a robust and versatile fluorophore scaffold but also highlights the importance of structural design in achieving selective *versus* broad-spectrum metal ion detection. The unique sensing behavior of SB-1, capable of responding to a range of biologically essential cations, and the specificity of SB-2 for Zn^2+^, opens new avenues for the development of smart, adaptable fluorescent probes for real-world applications in diagnostics, environmental monitoring, and cellular imaging.

## 2. Experimental section

### 2.1. Materials and instruments

All reagents and solvents used in this study were of analytical grade. The compounds 4-bromophenol, benzene-1,2-diamine, ethane-1,2-diamine, (4*a*,4*b*-dihydrophenanthren-9-yl)boronic acid, trifluoroacetic acid (TFA, Spectrochem), hexamethylenetetramine, and various metal salts (purchased from Merck India) were utilized directly without further purification. Solvents such as acetonitrile, ethanol, and *N*,*N*-dimethylformamide (DMF), all procured from Spectrochem, were purified according to standard laboratory procedures prior to use. Deionized water, further purified by double distillation, was used for preparing all aqueous solutions. Stock solutions of metal ions including Na^+^, K^+^, Mg^2+^, Al^3+^, Fe^2+^, Co^2+^, Hg^2+^, Ni^2+^, Mn^2+^, Sn^2+^, Pb^2+^, Cd^2+^, Cu^2+^, and Zn^2+^ were freshly prepared in deionized water. Column chromatography was performed using silica gel (60–120 mesh) as the stationary phase. NMR spectroscopic data were recorded on a JEOL JNM-ECZ-500R/M1 instrument (500 MHz for ^1^H and 125 MHz for ^13^C) using CDCl_3_ as the solvent, with tetramethylsilane (TMS) as the internal standard. High-resolution mass spectra (HRMS) were acquired on a Thermo Scientific Exactive Orbitrap mass spectrometer, and results are reported in mass-to-charge (*m*/*z*) ratios. UV-visible absorption measurements were carried out on a SHIMADZU UV-2600 spectrophotometer with a slit width of 2 nm. Fluorescence spectra were recorded using a PerkinElmer LS 6500 fluorescence spectrometer. Theoretical calculations were performed using density functional theory (DFT) at the B3LYP/6-31G* level with the Gaussian 09W software package.

### 2.2. UV-visible and fluorescence titration

UV-visible and fluorescence titration experiments for the compounds SB-1 and SB-2 were conducted using 1 mL of a 1 × 10^−5^ M solution prepared in DMSO (stock concentration: 1 × 10^−4^ M). Stock solutions of metal salts were prepared in water at a concentration of 1 × 10^−3^ M. For the titration studies, incremental additions of a 1 × 10^−4^ M aqueous solution of the respective metal ions were added to the probe solution. The changes in absorbance and fluorescence intensity were monitored to evaluate the binding interactions between the receptors and metal ions.

### 2.3. Synthesis and characterization

#### 2.3.1. Synthesis of 5-bromo-2-hydroxybenzaldehyde

**Scheme 1:**
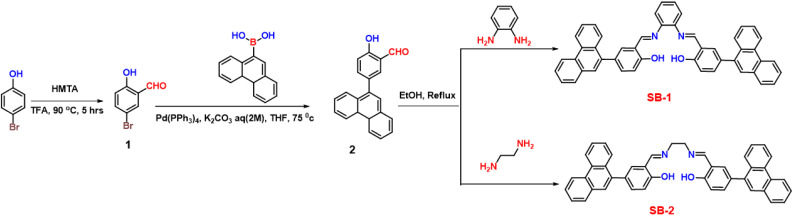
Synthesis of SB-1 and SB-2.

4-Bromophenol (10 g, 58 mmol) was dissolved in trifluoroacetic acid (100 mL), followed by the addition of hexamethylenetetramine (9.39 g, 67 mmol). The reaction mixture was refluxed at 90–100 °C for 5 hours. After completion, the mixture was cooled and stirred with 6 N HCl (100 mL) for 20 minutes. The product was then extracted using DCM and purified by column chromatography, yielding a yellow powder with 88% yield (Scheme 1).

#### 2.3.2. Synthesis of 5-(4*a*,4*b*-dihydrophenanthren-9-yl)-2-hydroxybenzaldehyde

In an oven-dried Schlenk flask, 5-bromo-2-hydroxybenzaldehyde (700 mg, 3.48 mmol) and phenanthren-9-ylboronic acid (850 mg, 3.83 mmol) were combined and dissolved in 50 mL of tetrahydrofuran (THF). A freshly prepared aqueous solution of potassium carbonate (1.4 g in 5 mL H_2_O) was added to the mixture. The resulting suspension was thoroughly degassed by bubbling nitrogen gas for 30 minutes. Following degassing, Pd(PPh_3_)_4_ (40 mg) was added as the catalyst under nitrogen atmosphere, and the reaction mixture was stirred under reflux at 75 °C for 24 hours. Reaction progress was monitored by TLC until complete consumption of the starting material was confirmed. After completion, the reaction mixture was cooled to room temperature and concentrated under reduced pressure. The residue was extracted using ethyl acetate and water, and the organic phase was subsequently washed with brine. The organic layer was dried over anhydrous sodium sulfate, and concentrated. Purification of the crude product was carried out by column chromatography on silica gel using a gradient of hexane and ethyl acetate as eluent. The desired product was obtained as an off-white solid in an isolated yield of 80.6% (837.9 mg). ^1^H NMR (500 MHz, CDCl_3_) *δ* 11.14 (s, 1H), 9.95 (s, 1H), 8.79 (d, *J* = 8.2 Hz, 1H), 8.73 (d, *J* = 8.3 Hz, 1H), 7.90 (d, *J* = 7.8 Hz, 1H), 7.85 (d, *J* = 8.2 Hz, 1H), 7.71 (dd, *J* = 7.4, 1.6 Hz, 3H), 7.68 (m, 2H), 7.64 (t, *J* = 6.9 Hz, 1H), 7.57 (t, *J* = 7.1 Hz, 1H), 7.15 (d, *J* = 9.2 Hz, 1H). ^13^C NMR (126 MHz, CDCl_3_) *δ* 197.1, 161.5, 139.2, 137.2, 135.2, 133.0, 131.9, 131.4, 131.2, 130.5, 129.1, 128.3, 127.5, 127.4, 127.2, 127.2, 126.8, 123.6, 123.1, 121.0, 118.1.

#### 2.3.3. Synthesis of SB-1

An oven-dried round-bottom flask was charged with 2-hydroxy-5-(phenanthren-9-yl)benzaldehyde (250 mg, 0.838 mmol), which was dissolved in ethanol. To this solution, benzene-1,2-diamine (50 mg, 0.461 mmol) was added, and the mixture was refluxed at 90 °C for 12 hours. As the reaction progressed, a solid precipitate gradually formed. Upon completion, the reaction mixture was allowed to cool and was further stored at low temperature for 30 minutes to enhance precipitation. The resulting orange solid was collected by vacuum filtration, washed thoroughly with cold ethanol, and dried under reduced pressure. Yield = 82%. ^1^H NMR (500 MHz) *δ* 13.19 (s, 2H), 9.07 (s), 8.94 (d, *J* = 8.2 Hz, 2H), 8.87 (d, *J* = 8.0 Hz, 2H), 8.03 (d, *J* = 7.5 Hz, 2H), 7.93 (d, *J* = 8.4 Hz, 2H), 7.90 (d, *J* = 6.9 Hz, 2H), 7.81 (s, 2H), 7.75–7.71 (m, 4H), 7.68 (d, *J* = 7.5 Hz, 2H), 7.62 (dd, *J* = 14.3, 5.7 Hz, 4H), 7.50 (d, *J* = 3.4 Hz, 2H), 7.42 (d, *J* = 2.5 Hz, 2H), 7.17 (d, *J* = 8.6 Hz, 2H). ^13^C NMR (500 MHz, DMSO) *δ* 164.6, 160.5, 142.8, 137.6, 135.4, 134.8, 133.4, 131.6, 131.3, 130.9, 130.8, 129.1, 128.4, 127.8, 127.6, 127.3, 126.7, 126.7, 123.9, 123.3, 120.3, 120.0, 117.4, 117.1. HRMS (EI): calculated for C_48_ H_32_N_2_O_2_ [M–H]^+^, *m*/*z* 669.2542, found *m*/*z* 669.2540.

#### 2.3.4. Synthesis of SB-2

Compound SB-2 was prepared following a procedure similar to that used for compound SB-1 replacing the benzene-1,2-diamine with ethylenediamine. The quantities involved and characterization data are as follows. 2-hydroxy-5-(phenanthren-9-yl)benzaldehyde (250 mg, 0.838 mmol), ethane-1,2-diamine (27.69 mg, 0.461 mmol) and ethanol (50 mL) yield = 89%. ^1^H NMR (400 MHz, DMSO) *δ* 13.61 (s, 2H), 8.93 (d, *J* = 8.3 Hz, 2H), 8.85 (s, 2H), 8.73 (s, 2H), 8.00 (d, *J* = 7.5 Hz, 2H), 7.86 (d, *J* = 8.2 Hz, 2H), 7.76 (s, 2H), 7.74–7.67 (m, 5H), 7.65 (d, *J* = 2.1 Hz, 3H), 7.59 (t, *J* = 7.6 Hz, 2H), 7.51 (d, *J* = 8.4 Hz, 2H), 7.08 (s, 2H), 4.00 (s, 4H). ^13^C NMR (101 MHz, DMSO) *δ* 167.5, 160.8, 137.7, 134.3, 133.2, 131.6, 130.9, 130.7, 130.6, 129.8, 129.1, 127.7, 127.6, 127.4, 127.3, 126.6, 123.9, 123.2, 118.9, 117.2, 59.2. HRMS (EI): calculated for C_44_ H_32_N_2_O_2_ [M–H]^+^, *m*/*z* 621.2542, found *m*/*z* 620.2547.

## 3. Results and discussions

SB-1 and SB-2, two Schiff base-based chemosensors, were synthesized *via* a simple condensation reaction between 5-(4*a*,4*b*-dihydrophenanthren-9-yl)-2-hydroxybenzaldehyde and either benzene-1,2-diamine (for SB-1) or ethane-1,2-diamine (for SB-2) in ethanol under reflux conditions, as depicted in Scheme 1. Following the reaction, the pure products were isolated as red solids by washing with cold ethanol and diethyl ether. The compounds were subsequently purified and characterized using a combination of ^1^H and ^13^C NMR spectroscopy, along with high-resolution ESI-HRMS to confirm their molecular structures. In the ^1^H NMR spectrum of SB-1, a prominent signal at *δ* = 9.06 ppm was attributed to the proton of the extended phenyl conjugation, consistent with the expected structure. For SB-2, the imine proton (HC = N) appeared at *δ* = 8.73 ppm, showing a slight upfield shift compared to SB-1, indicative of structural variations in the imine linkage. The ^13^C NMR spectrum revealed the imine carbon in SB-1 at *δ* = 160.4 ppm, whereas SB-2 showed a corresponding signal at *δ* = 160.8 ppm, further confirming the subtle differences in their molecular environments. The NMR spectra for both compounds displayed well-defined chemical shifts and integration values, supporting the successful formation of the intended Schiff base structures. The identities of SB-1 and SB-2 were further validated by ESI-HRMS analysis. The molecular ion for SB-1 was observed at *m*/*z* 669.2540, while SB-2 displayed a peak at *m*/*z* 621.2547, corresponding to their respective molecular weights. These results, in combination with the NMR data, explicitly confirm the successful synthesis of the Schiff base chemosensors and their expected molecular compositions.

### 3.1. Photophysical properties

After successfully isolating pure form of SB-1 and SB-2, their optical properties were thoroughly investigated using UV-Vis absorption and fluorescence spectroscopy in DMSO solutions. The absorption spectrum of SB-1 exhibited three distinct peaks at 260, 300, and 365 nm, which can be attributed to the electronic transitions within the aromatic system, the azomethine (–CH=N–) bond, and the hydroxyl (–OH) group. These absorption features are indicative of π–π and n–π transitions that are typical for Schiff base compounds containing conjugated structures. On the other hand, SB-2 displayed two major absorption peaks at 260 and 300 nm, suggesting a lack of additional conjugation from the phenyl group present in SB-1. The absence of the 365 nm absorption peak in SB-2 supports the idea that the phenanthrene unit in SB-1 contributes significantly to its enhanced absorption characteristics, likely due to the extended conjugation that facilitates additional π–π* transitions. These absorption profiles are summarized in Fig. S9. In terms of fluorescence properties, both SB-1 and SB-2 demonstrated relatively weak emission in DMSO solution. This low fluorescence intensity can be attributed to the well-known photon-induced electron transfer (PET) process commonly observed in Schiff base derivatives, thus quenching the fluorescence. In addition to our photophysical investigations, we measured the fluorescence quantum yields of SB-1 and SB-2 to further validate their emission characteristics. SB-1 exhibited a moderate quantum yield of 0.04, whereas SB-2 showed a significantly lower value of 0.01. These results are in good agreement with previously reported values for similar Schiff base systems and reinforce the conclusion that SB-2 possesses inherently weaker emissive properties compared to SB-1, which can be explained by the presence of the phenyl unit, which makes the system rigid. Overall, these optical studies underscore the significant role of molecular structure in governing the photophysical properties of Schiff base-based sensors. The differences observed in the absorption and fluorescence behaviour of SB-1 and SB-2 highlight the potential for tuning optical properties by modifying the ligand environment, which could be crucial for improving their performance in sensing applications.

### 3.2. UV-Vis absorption and fluorescence titration studies

**Fig. 1:**
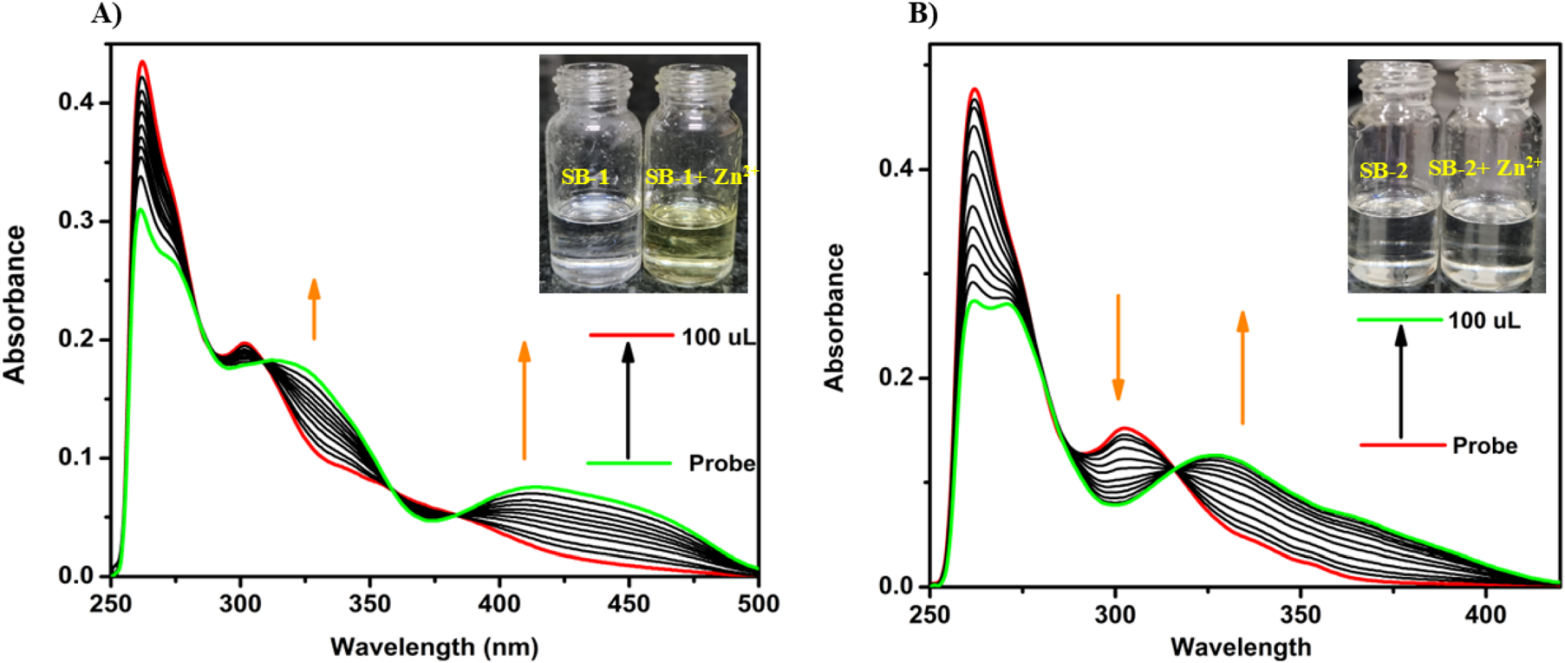
UV-visible absorbance spectra of (A) SB-1 (left) and (B) SB-2 (right) in DMSO before and after the addition of Zn^2+^ ions. (Inset: images of the solutions under ambient light).

**Scheme 2:**
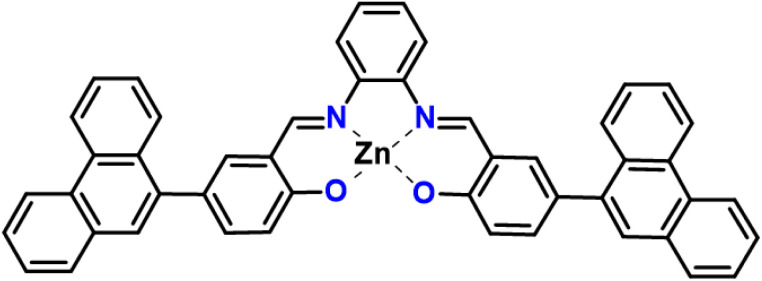
Nature of metal binding of Schiff bases.

UV-visible and fluorescence spectroscopy were employed to investigate the metal ion sensing capabilities of SB-1 and SB-2. The UV-visible absorption spectra were monitored in DMSO upon the addition of various metal ions, including Na^+^, K^+^, Mg^2+^, Al^3+^, Fe^2+^, Co^2+^, Hg^2+^, Ni^2+^, Mn^2+^, Sn^2+^, Pb^2+^, Cd^2+^, Cu^2+^, and Zn^2+^. Significant spectral changes were observed for SB-1 in the presence of Na^+^, K^+^, Mg^2+^, Cu^2+^, and Zn^2+^ ions, as illustrated in Fig. 1, and S10–S12. In contrast, SB-2 exhibited noticeable UV-spectral changes primarily only towards Cu^2+^ and Zn^2+^ ions. For SB-1, the addition of Zn^2+^ ions resulted in a gradual increase in absorption bands at 415 and 315 nm, while absorption intensities at 365 and 295 nm simultaneously decreased. Similarly, Na^+^, K^+^, Mg^2+^, and Cu^2+^ ions influenced the absorption spectrum in a comparable manner. Upon addition of Zn^2+^ to SB-1, a distinct increase in absorption bands at 415 and 315 nm was observed, accompanied by a decrease in intensities at 365 and 295 nm. Comparable spectral alterations were noted in the presence of Na^+^, K^+^, Mg^2+^, and Cu^2+^, suggesting similar binding interactions. SB-2, on the other hand, showed a subtle increase at 325 nm and a decrease at 300 nm upon Zn^2+^ addition, although the spectral response was significantly weaker than that of SB-1. A notable visual colour change from colourless to yellow was observed upon adding Zn^2+^ ions to the SB-1 solution, attributed to the formation of a new absorption band in the bathochromic region (∼415 nm) (Fig. 1 and S10–S12). This result demonstrates that SB-1 can detect Zn^2+^ ions in aqueous solutions with the naked eye, even in the presence of other competing metal ions. The emergence of this bathochromic band is likely due to intra-ligand charge transfer, redshifted upon strong metal chelation. In the case of Cu^2+^, SB-1 displayed enhanced absorption at 315 and 415 nm, while SB-2 exhibited a peak shift centered around 320 nm. Competitive experiments, involving an excess of other metal ions, confirmed the selective sensitivity of SB-1 toward Na^+^, K^+^, Cu^2+^, Mg^2+^ and Zn^2+^, which induced new bands at 400 and 300 nm and a concurrent decrease at 365 and 290 nm. Meanwhile, SB-2 remained largely inert to these metal ions, underscoring its comparatively narrower sensing profile (Scheme 2).

**Fig. 2:**
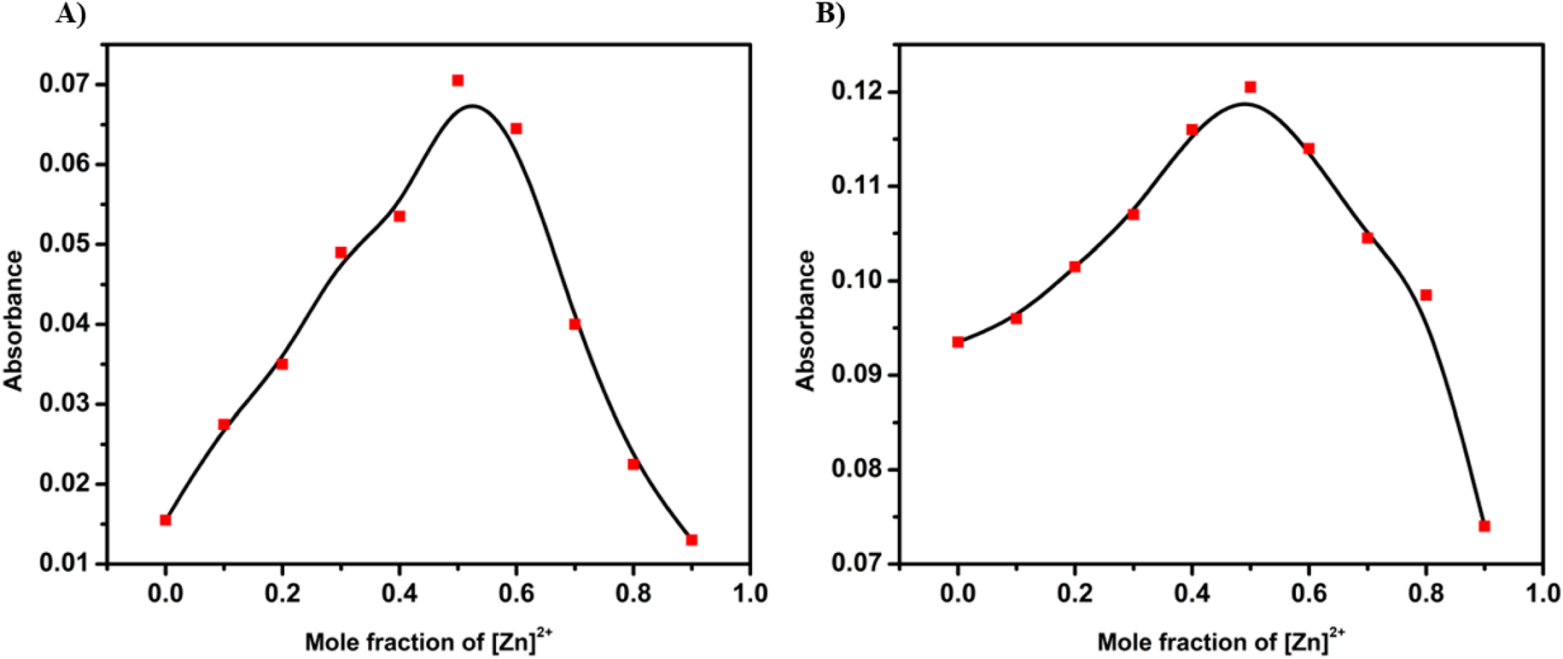
Job's plot according to the method of continuous variations, indicating the 1 : 1 stoichiometry for compounds (A) SB-1 (A) and (B) SB-2 (B) with Zn^2+^ions.

The metal-binding behaviour of SB-1 and SB-2 was further elucidated through the identification of isosbestic points in their UV-visible absorption spectra. SB-1 displayed three well-defined isosbestic points upon interaction with selected metal ions, suggesting a clean interconversion between the free ligand and metal-bound forms. Specifically, Zn^2+^ and Cu^2+^ induced isosbestic transitions at 380, 360, and 310 nm, while Na^+^, K^+^, and Mg^2+^ triggered similar transitions at 380, 350, and 320 nm, respectively. In contrast, SB-2 showed only two isosbestic points, with Cu^2+^ producing transitions at 315 and 290 nm, and Zn^2+^ at 315 and 285 nm. These observations indicate fast and discrete complexation events, reflecting the formation of well-defined metal–ligand species. The clear visual color change observed in SB-2, particularly upon binding Zn^2+^ and Cu^2+^, highlights its potential as a practical colorimetric sensor for these ions, with detectable changes even in competitive environments (Fig. S13). To quantify these interactions, association constants were determined using the Benesi–Hildebrand method. Among all tested ions, Zn^2+^ showed the highest binding affinity for SB-1, while Cu^2+^ displayed the strongest interaction with SB-2. In contrast, Mg^2+^ exhibited the weakest binding to SB-1, and Zn^2+^ showed the lowest affinity for SB-2 (Table S1). The overall binding strength followed the order Zn^2+^ > K^+^ > Na^+^ > Cu^2+^ > Mg^2+^ for SB-1 and Cu^2+^ > Zn^2+^ for SB-2. The detection limits (LOD) for each sensor-ion pair were calculated using the formula LOD = 3*σ*/*S*, where *σ* represents the standard deviation of the blank and *S* denotes the slope of the calibration curve derived from titration data. Remarkably, SB-2 exhibited the highest sensitivity toward Cu^2+^, with an LOD of 0.05 μM, while SB-1 also demonstrated strong detection capabilities with a Cu^2+^ LOD of 0.10 μM (Table S1). In addition, other metal ions such as Zn^2+^, K^+^, Na^+^, and Mg^2+^ also demonstrated detection limits in μM with both SB-1 and SB-2, highlighting their broad applicability. Absorbance values for both chemosensors plateaued after the addition of 1.0 equivalent of their respective target ions, confirming the formation of 1 : 1 ratio of metal and Schiff base complexes. Further insights into the binding stoichiometry were obtained from Job's plot analyses, which supported the proposed 1 : 1 ligand-to-metal complexation ratio for the most responsive ions. Upon adding metal ions to the receptors SB-1 and SB-2, the absorption maxima at 420 nm were evaluated, to determine the binding stoichiometry, varying mole ratios of metal ions to receptors (0.1, 0.2, 0.3, 0.4, 0.5, 0.6, 0.7, 0.8, and 0.9). The Job's plot analysis, as illustrated in Fig. 2, and S15, revealed that the maximum absorption occurred at a 0.5 mole fraction for both SB-1 and SB-2 with Zn^2+^ ions, indicating a 1 : 1 binding stoichiometry. A similar trend was observed for other metal ions, including Cu^2+^, Na^+^, K^+^, and Mg^2+^, as shown in Fig. S14, and S16. These results underscore the effectiveness of SB-1 and SB-2 as selective and sensitive probes for transition and alkali/alkaline earth metal ions.

**Fig. 3:**
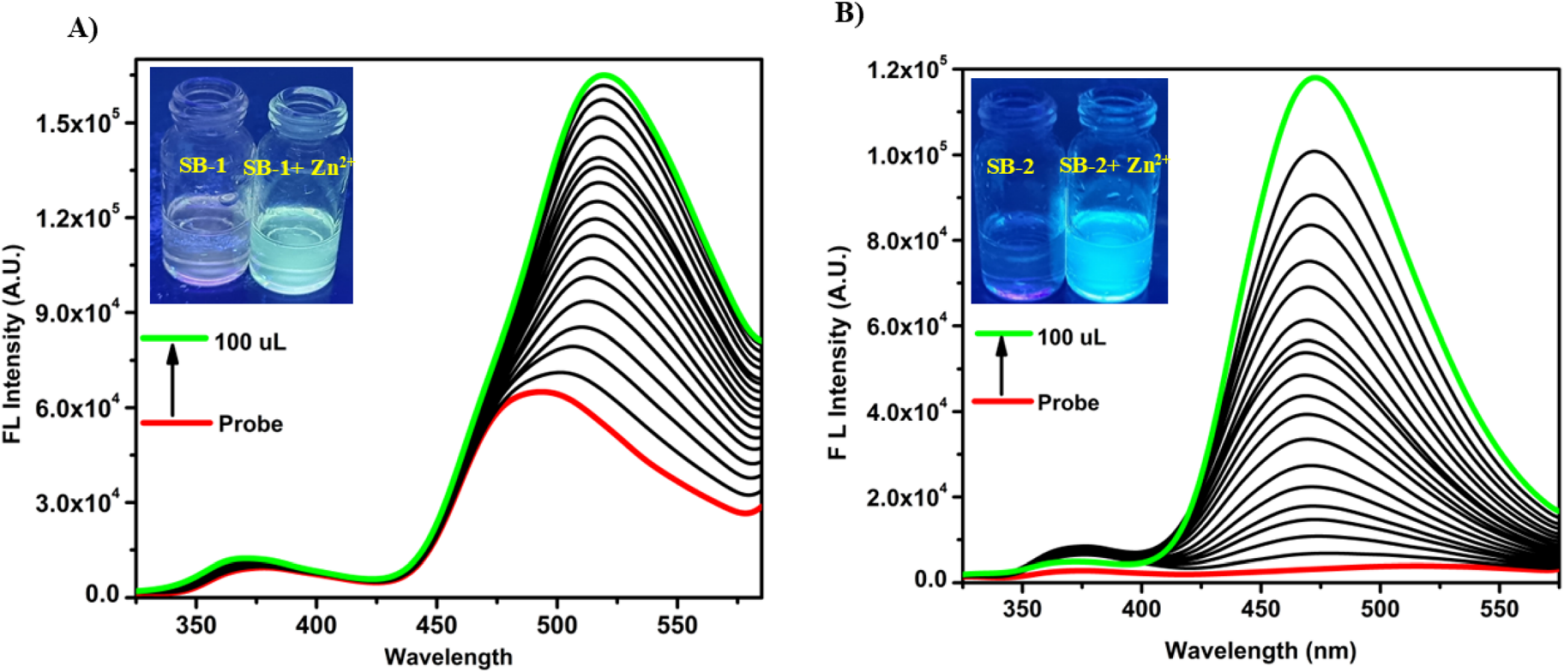
Emission spectral changes of (A) SB-1 (left, 10^−5^ M) and (B) SB-2 (right, 10^−5^ M) upon the addition of Zn^2+^ ions in DMSO. (Inset: photographs captured in DMSO under UV light).

To gain deeper insights into the fluorescence behavior and metal ion sensing potential of SB-1 and SB-2, detailed fluorescence titration experiments were conducted under standardized conditions. The selectivity and sensitivity of both chemosensors were evaluated using a comprehensive set of metal ions, including Na^+^, K^+^, Mg^2+^, Al^3+^, Fe^2+^, Co^2+^, Hg^2+^, Ni^2+^, Mn^2+^, Sn^2+^, Pb^2+^, Cd^2+^, Cu^2+^, and Zn^2+^. Initially, both SB-1 and SB-2 exhibited a very weak fluorescence emission band centered at ∼490 nm when excited at 350 nm in DMSO, indicating their non-emissive nature in the free state due to possible non-radiative decay pathways, such as –C=N– isomerization and PET (photoinduced electron transfer). Upon gradual addition of Zn^2+^ ions to a 10^−5^ M solution of SB-1 and SB-2, a significant “*turn-on*” fluorescence response was observed, with emission intensity progressively increasing with gradual addition of Zn^2+^ ions (Fig. 3). The observed fluorescence enhancement is attributed to the suppression of non-radiative pathways such as C=N isomerization, PET, and ESIPT (excited-state intramolecular proton transfer) processes, which are otherwise active in the free ligands. Coordination of metal ions with the imine nitrogen and phenolic oxygen moieties stabilizes the excited state and restricts conformational flexibility, thereby allowing efficient radiative decay. These mechanisms are well-supported by literature precedent for Schiff-base type fluorophores. Similarly, SB-1 also showed fluorescence enhancement in the presence of Na^+^, K^+^, and Mg^2+^ ions, although the intensity was lower compared to the Zn^2+^ induced emission, indicating a broader sensing profile for SB-1 (Fig. S24). On the other hand, SB-2 exhibited a vivid yellow fluorescence exclusively in the presence of Zn^2+^ ions, while no significant change in emission spectra was observed upon the addition of Na^+^, K^+^, or Mg^2+^ ions, thereby confirming its high selectivity toward Zn^2+^. Furthermore, we evaluated the fluorescence quantum yields of SB-1 and SB-2 in the presence of various metal ions to better understand their sensing efficiencies. SB-1 exhibited the highest quantum yield upon coordination with Mg^2+^ (0.32), followed by Zn^2+^ (0.28), Na^+^ (0.12), and K^+^ (0.10), indicating a broad yet differential fluorescence enhancement across multiple biologically relevant metal ions. In contrast, SB-2 demonstrated a pronounced *turn-on* response specifically with Zn^2+^, showing a significantly higher quantum yield of 0.54. Interestingly, no notable fluorescence enhancement or spectral shift was observed for either SB-1 or SB-2 upon exposure to other tested metal ions, even at higher concentrations. This selective response underscores the high specificity of SB-1 and SB-2 toward certain metal ions, particularly Zn^2+^. Visual confirmation of this fluorescence enhancement was evident under UV light (365 nm), where the initially non-fluorescent SB-1 solution emitted a bright yellow fluorescence upon the addition of Na^+^, K^+^, Mg^2+^, or Zn^2+^ ions. Among the two receptors, SB-1 emerged as the more efficient fluorescent sensor for Zn^2+^, as evidenced by its higher fluorescence enhancement and broader reactivity towards additional biologically and environmentally relevant ions like Na^+^, K^+^, and Mg^2+^ (Fig. S24). This highlights its potential utility for multianalyte detection. We have systematically investigated the influence of different counter anions (such as Cl^−^, NO_3_^−^, SO_4_^2−^, and AcO^−^) associated with Zn^2+^ on the fluorescence response of SB-2. The results revealed that the nature of the anion had negligible impact on the fluorescence behavior of SB-2. This suggests that the observed fluorescence enhancement is primarily driven by the coordination of the Zn^2+^ cation with the probe, and not significantly affected by its accompanying counter anion. Such stability toward varying anionic environments further demonstrates the robustness and reliability of SB-2 as a selective Zn^2+^ sensor, even in complex media containing different zinc salts.

**Fig. 4:**
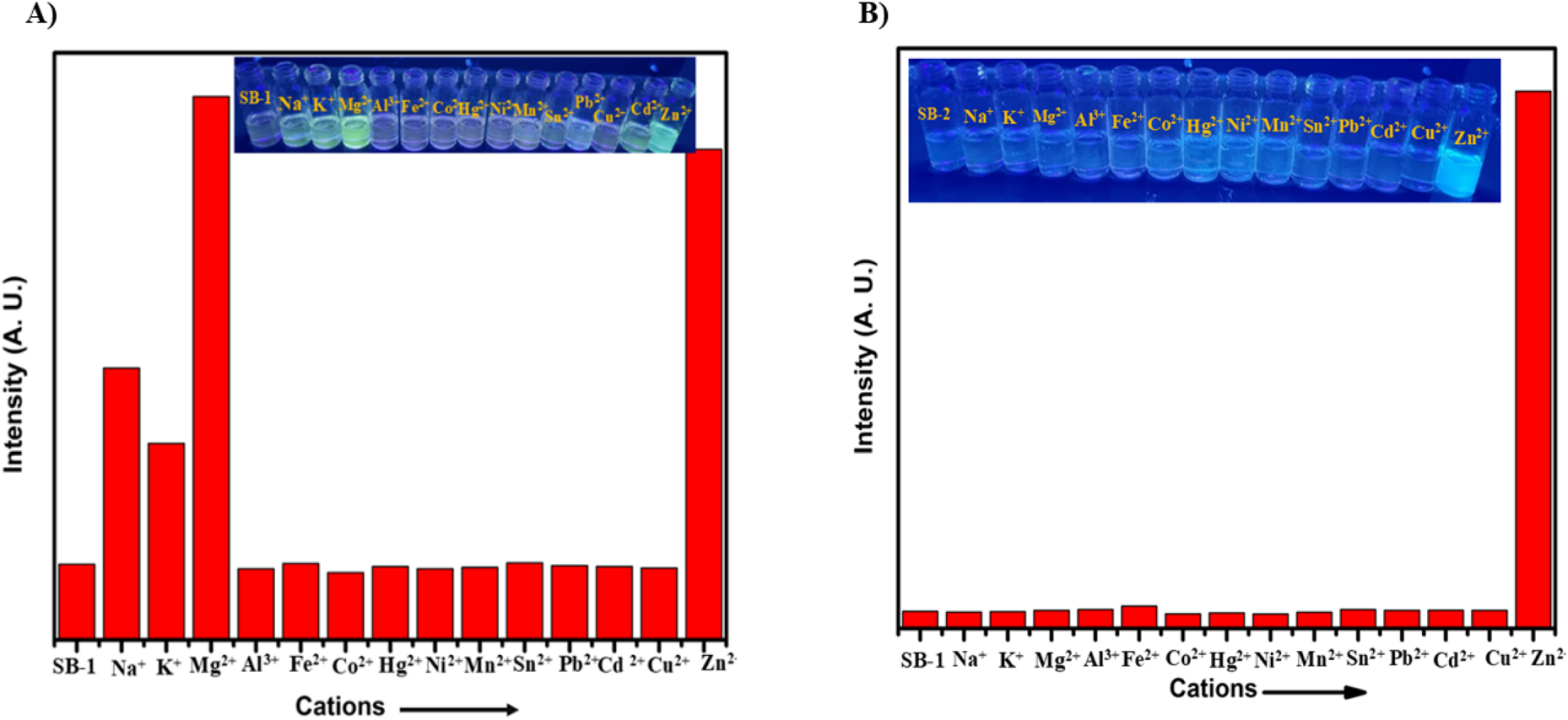
Selectivity analysis of SB-1 (A) and SB-2 (B) (1 × 10^−5^ M) toward various metal ions. (Inset: photographs captured in DMSO under UV light).

**Fig. 5:**
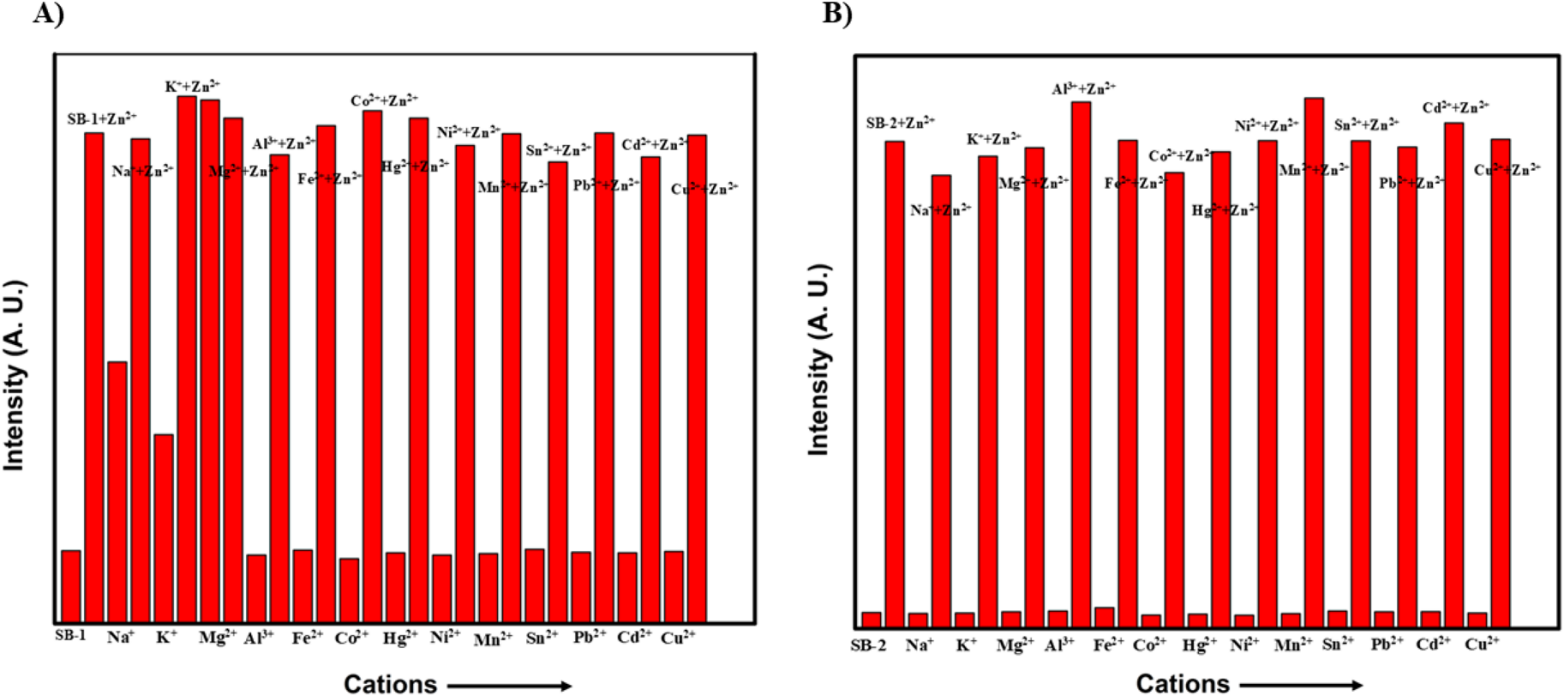
Examination of competitive studies of SB-1 (A) and SB-2 (B) (1 × 10^−5^ M) towards various metal ions. The competitive binding ability of CS-1 and CS-2 (1 × 10^−5^ M) in the presence of other interfering metal ions and emission band was monitored at 520 nm.

Achieving high selectivity and sensitivity in the presence of multiple metal ions is a key requirement for the practical application of fluorescent chemosensors. To explore this, SB-1 and SB-2 were systematically examined for their fluorescence responses toward a panel of biologically and environmentally relevant metal ions, including Na^+^, K^+^, Mg^2+^, Al^3+^, Fe^2+^, Co^2+^, Hg^2+^, Ni^2+^, Mn^2+^, Sn^2+^, Pb^2+^, Cd^2+^, Cu^2+^, and Zn^2+^. Fluorescence titration experiments were conducted using 10^−5^ M solutions of SB-1 and SB-2 in DMSO to monitor changes in emission behaviour upon the addition of these metal ions (Fig. 4). The results showed that SB-1 exhibited a clear and selective fluorescence enhancement in the presence of Na^+^, K^+^, Mg^2+^, and Zn^2+^ ions. Notably, the fluorescence remained unaltered or only marginally affected when other metal ions were introduced, indicating strong specificity for these four analytes. This response was both rapid and stable, suggesting efficient coordination between SB-1 and these target ions. In contrast, SB-2 displayed highly selective fluorescence activation exclusively with Zn^2+^ ions. No observable enhancement was detected with Na^+^, K^+^, Mg^2+^, or any other tested metal ions, underscoring SB-2's strong preference and exceptional selectivity for Zn^2+^. This behavior was maintained even in the presence of excess amounts of potentially interfering ions, confirming the sensor's discrimination capability under competitive conditions (Fig. 5). Further, competitive studies of SB-1 and SB-2 with Zn^2+^ in the presence of other metal ions and results strongly suggested that the emission intensities remained stable, indicating saturation of binding interactions and does not influence the other metal ions. Together, these results highlight SB-1 as a versatile sensor capable of detecting multiple metal ions (Na^+^, K^+^, Mg^2+^, and Zn^2+^), while SB-2 stands out as a highly selective and sensitive probe for Zn^2+^. These findings clearly indicate that, even in the presence of other interfering metal ions, the SB-1 has high selectivity for Na^+^, K^+^, Mg^2+^, Zn^2+^, and SB-2 for Zn^2+^ ions. Such selective recognition profiles make these chemosensors promising tools for real-time metal ion detection in complex matrices.

### 3.3. ^1^H NMR titration studies

**Fig. 6:**
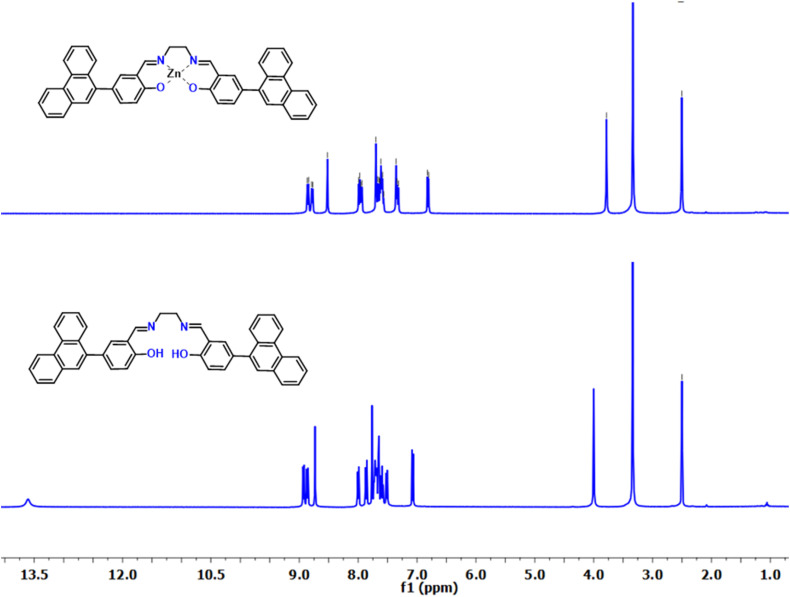
Compared ^1^HNMR of SB-2 and SB-2 + Zn^2+^ (NMR titration in DMSO-*d*_6_).

To further investigate the coordination behavior of SB-1 and SB-2 with Zn^2+^ ions, ^1^H NMR titration studies were carried out in DMSO-*d*_6_. Upon gradual addition of Zn^2+^ to SB-2, noticeable changes were observed in the chemical shifts of key proton signals (Fig. 6), and S25 indicating complex formation. The most prominent shift was seen in the imine proton (–CH=N–), which moved downfield, reflecting its direct involvement in metal coordination. Concurrently, the disappearance of the phenolic –OH proton resonance at *δ* = 13.60 ppm strongly suggested deprotonation of the hydroxyl group upon binding to Zn^2+^. These spectral alterations confirm that Zn^2+^ coordinates with SB-2 through both the imine nitrogen and phenolic oxygen atoms. The overall downfield migration of aromatic proton signals further supports the formation of a stable metal–ligand complex, likely accompanied by electronic reorganization within the aromatic system. Similar spectral behavior was observed during the titration of SB-1 with Zn^2+^, reinforcing a comparable binding mode for both receptors. These findings, derived from ^1^H NMR evidence, clearly demonstrate the strong and selective interaction between the ligands and Zn^2+^ ions.

### 3.4. DFT theoretical studies

**Fig. 7:**
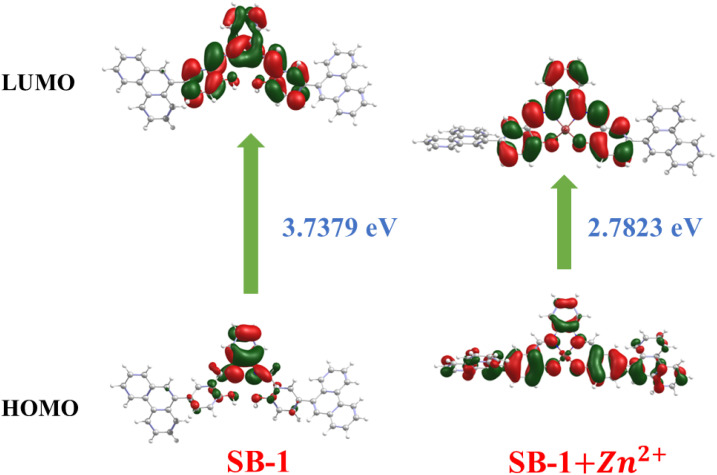
Selected MOs of SB-1 and SB-1 + Zn^2+^ (not to scale; isocontour value = 0.02).

Molecular orbital analysis provides useful information about the electronic structure and can be used to determine how receptors interact with the metal ions. Density functional theory (DFT) calculations for all compounds were performed using the Gaussian 09W program package. These calculations were carried out at the B3LYP level^19^ for C, H, N, and O atoms, allowing for a comprehensive understanding of the electronic structure and metal-binding properties of the probes. The lowest unoccupied molecular orbital (LUMO), which shows the charge transfer from the aromatic unit to the imine moiety, is highly located on the entire π-moiety except the phenanthrene ring in SB-1 and while in SB-2, it extends over the entire molecule except for the ethane segment. The highest occupied molecular orbital (HOMO) is concentrated on the π-moiety of the diamino benzene unit in SB-1 and on one side of the molecule in SB-2 (Fig. 7 and S26). According to DFT calculations, the HOMO–LUMO energy gap of SB-1 and SB-2 was 3.74 and 4.38 eV, respectively which correlates with the experimental value. Upon the introduction of Zn^2+^ ions to the receptors, the HOMO in SB-1 becomes localized over the entire π-moiety, whereas in SB-2, it is concentrated on one side of the molecule. The LUMO, on the other hand, is distributed across the π-system, excluding the phenanthrene ring, with minimal contribution from Zn^2+^ ions. This interaction leads to a significant reduction in the HOMO–LUMO energy gap, decreasing to 2.78 eV for SB-1 and 3.56 eV for SB-2. This reduction correlates with the emergence of a new absorption band (∼440 nm) in the UV-visible spectrum of SB-1 + Zn^2+^ and SB-2 + Zn^2+^. Fig. 7 illustrates the molecular structure and energy gap (*E*_g_) of SB-1 and its Zn^2+^ complex, while Fig. S26 presents the same for SB-2 and SB-2 + Zn^2+^. These findings strongly support the idea that Zn^2+^ coordination with the receptors modulates intraligand charge transfer, leading to distinct photophysical changes. The colorimetric response of SB-1 and SB-2 upon zinc ion binding is well substantiated by absorption spectral shifts, with the observed bathochromic shift serving as direct evidence of the narrowing HOMO–LUMO energy gap.

### 3.5. Reversibility studies

**Fig. 8:**
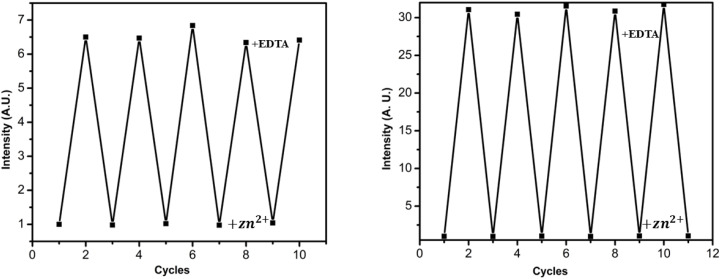
Reversibility studies of SB-1 (left) and SB-2 (right) were conducted by monitoring fluorescence intensity at 520 nm over five regeneration cycles, with alternating additions of Zn^2+^ followed by EDTA.

For practical applications, a chemosensor must exhibit not only high sensitivity and selectivity but also excellent reversibility. To assess the regenerative capability of SB-1 and SB-2, reversibility experiments were conducted using EDTA as a strong competing ligand. Upon the addition of 10 equivalents of EDTA to the SB-1·Zn^2+^ and SB-2·Zn^2+^ complexes in DMSO, a rapid quenching of fluorescence was observed, restoring the emission profile of the free receptors (Fig. 8). This clearly indicates that EDTA effectively sequesters Zn^2+^ from the sensor–metal complex due to its stronger binding affinity, thereby regenerating the original receptor. These results confirm that both SB-1 and SB-2 exhibit excellent reversibility and can be efficiently recycled through simple EDTA treatment. Notably, the receptors maintained their structural and optical integrity over multiple cycles, with minimal variation in absorption and emission intensity observed across at least five regeneration cycles (Fig. 8). This high stability and reversibility highlight the practical utility of SB-1 and SB-2 as reusable fluorescent sensors for Zn^2+^ detection Fig. 8.

## 4. Conclusion

We developed and characterized two Schiff base-based chemosensors, SB-1 and SB-2, using NMR and ESI-HRMS. Their sensing properties in DMSO were studied *via* UV-visible and fluorescence spectroscopy. SB-1 selectively responded to Na^+^, K^+^, Mg^2+^, and Zn^2+^ ions with a distinct colour change and fluorescence enhancement, while SB-2 showed high fluorescence selectivity for Zn^2+^ alone. These responses are attributed to intraligand charge transfer and suppression of –C=N– isomerization, PET, and ESIPT processes upon metal coordination. Job's plot confirmed 1 : 1 binding stoichiometry, and ^1^H NMR titrations supported the proposed coordination *via* imine nitrogen and phenolic oxygen. Both sensors demonstrated excellent reversibility with EDTA and maintained stability over multiple cycles. SB-1 showed superior sensitivity for Zn^2+^, with lower detection limits and higher binding constants than SB-2. Overall, SB-1 and SB-2 are effective, selective, and reversible chemosensors for biologically relevant metal ions.

## Conflicts of interest

The authors declare no competing financial interest.

## Supplementary Material

RA-015-D5RA03617H-s001

## Data Availability

The data underlying this study are available in the published article and its SI. ^1^H NMR, ^13^C NMR, HRMS, and characterization data for all molecules, photophysical data, DFT and TD-DFT results (PDF). See DOI: https://doi.org/10.1039/d5ra03617h.
