# Supplementary material for: Tuning metal ion affinity in acyclic phenanthrene schiff bases: comparative study of ethylene and phenylene linkers
Source: RSC Adv. 2025 Aug 11;15(35):28481–91. doi: 10.1039/d5ra03617h (PMC12377188; doi:10.1039/d5ra03617h)
Supplement: RA-015-D5RA03617H-s001 [file RA-015-D5RA03617H-s001.pdf]

Supplementary Information (SI) for RSC Advances.
This journal is © The Royal Society of Chemistry 2025

Tuning Metal Ion Affinity in Acyclic Phenanthrene Schiff Bases: Comparative Study of Ethylene and Phenylene Linkers

Haritha C[a], Swathi M[a] and Chinna Ayya Swamy P[a*]

[a]Main group Organometallics Optoelectronic Materials and Catalysis lab, Department of Chemistry, National Institute of Technology, Calicut, India-673601.

Corresponding author: swamy@nitc.ac.in

Table of contents

1. Characterisation data S2-S5
2. Photophysical Properties S6-S14
3. Detection limits and Association constants of the synthesised probes S14
4. ^{1}H NMR titration data S15
5. DFT computational data S16-S27

Characterisation data

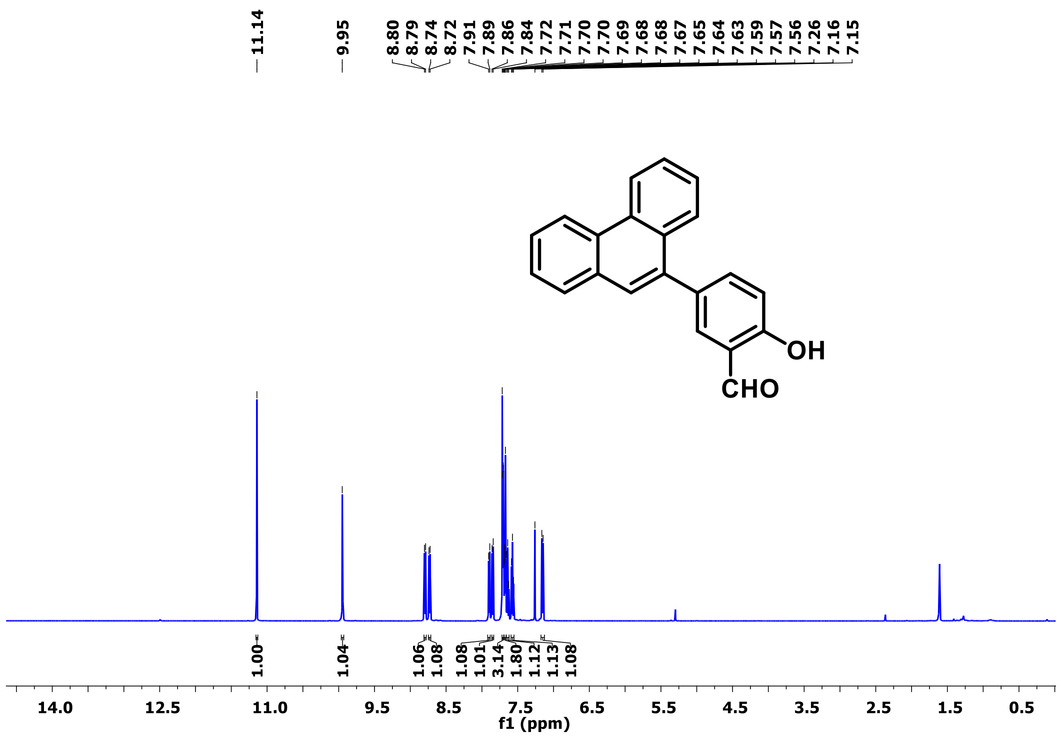

Figure S1. ^{1}H NMR of **2** (500 MHz, **CDCl₃**, RT)

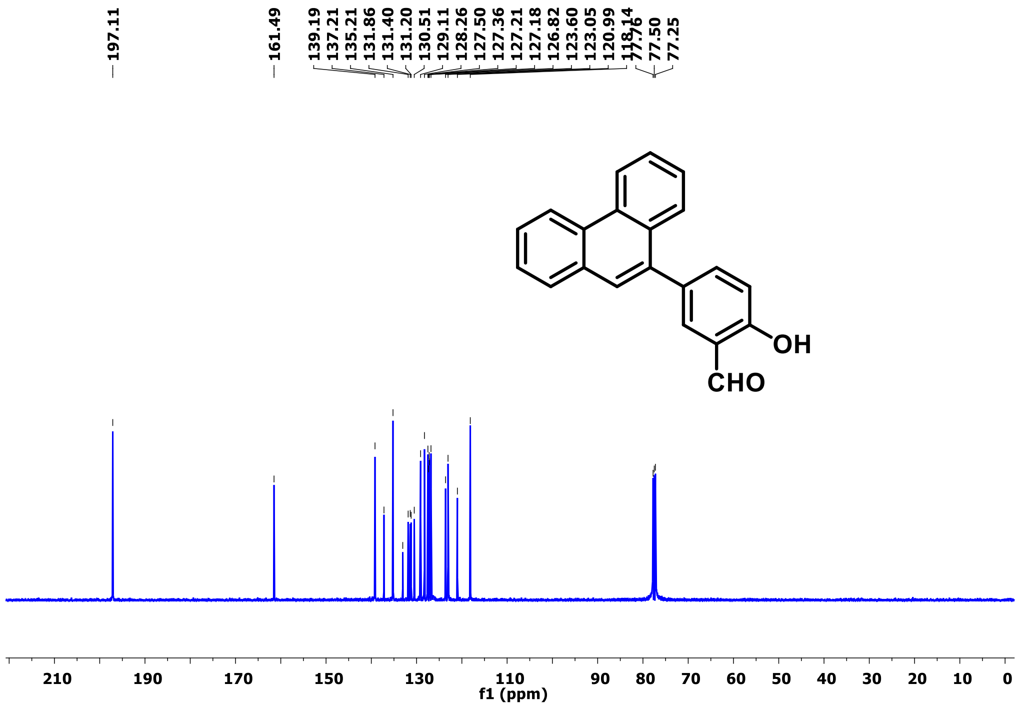

Figure S2. ^{13}C{^{1}H} NMR of **2** (**CDCl₃**, 126 MHz, RT).

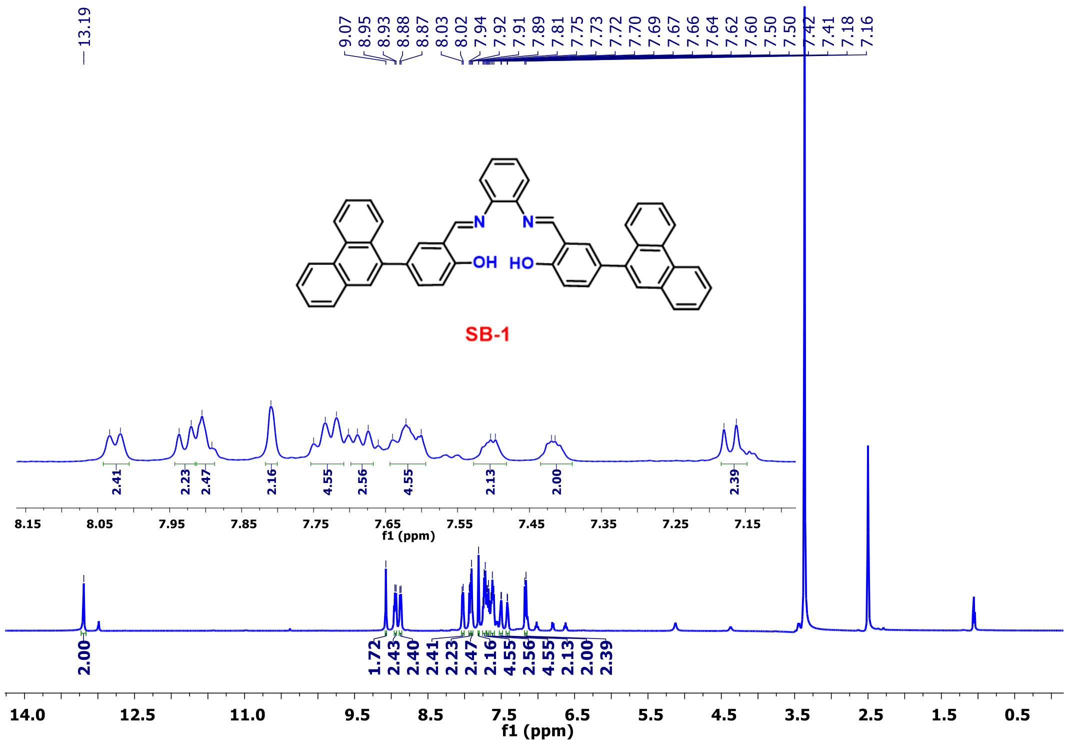

Figure S3. ^{1}H NMR of **SB-2** (500 MHz, **DMSO-d₆**, RT)

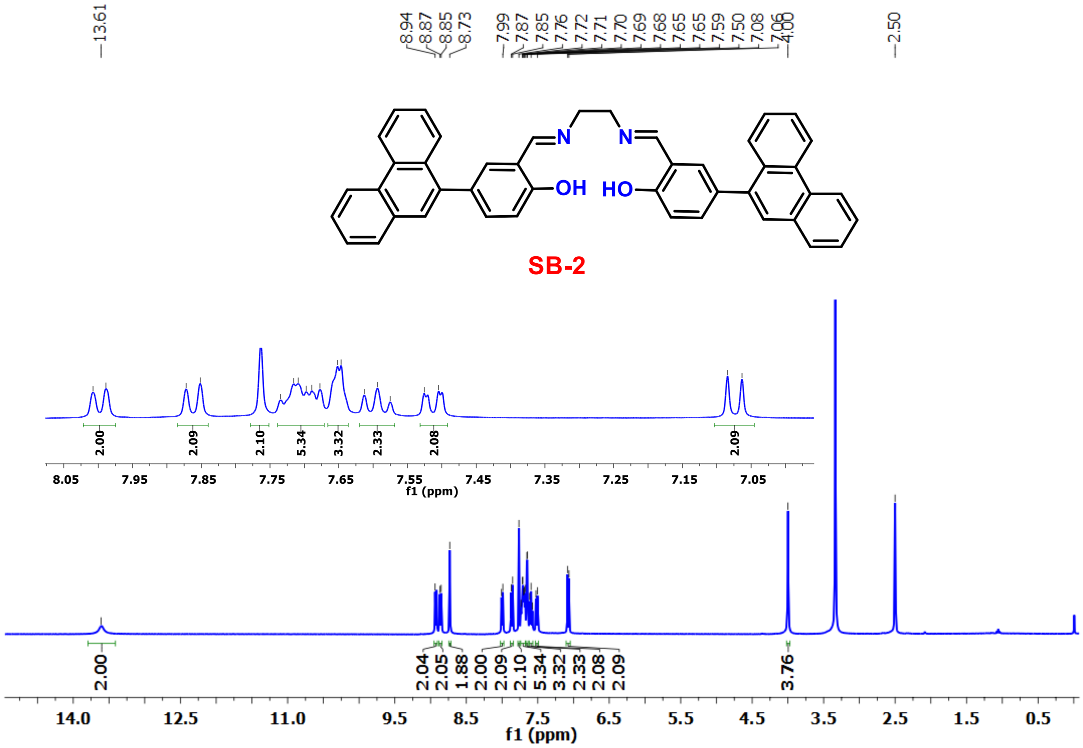

Figure S4. ^{1}H NMR of **SB-2** (500 MHz, **DMSO-d₆**, RT)

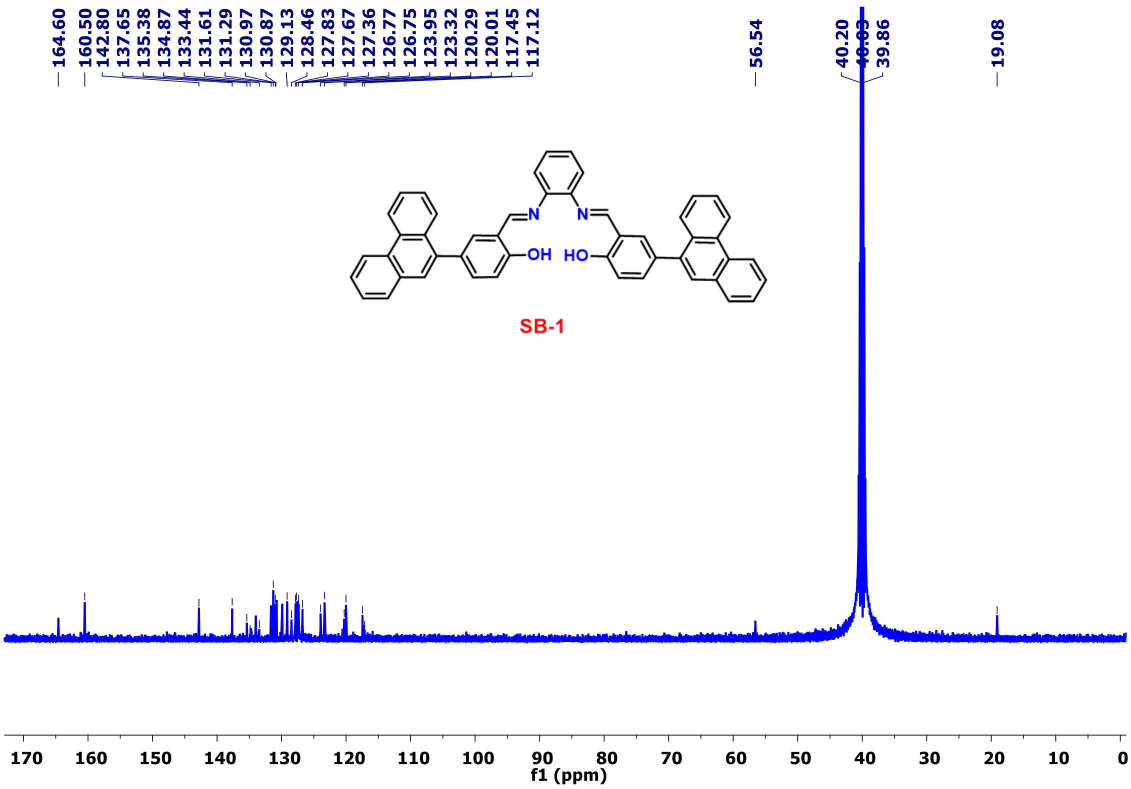

Figure S5. $^{13}C\{^{1}H\}$ NMR of **SB-1** (**DMSO-d6**, 126 MHz, RT).

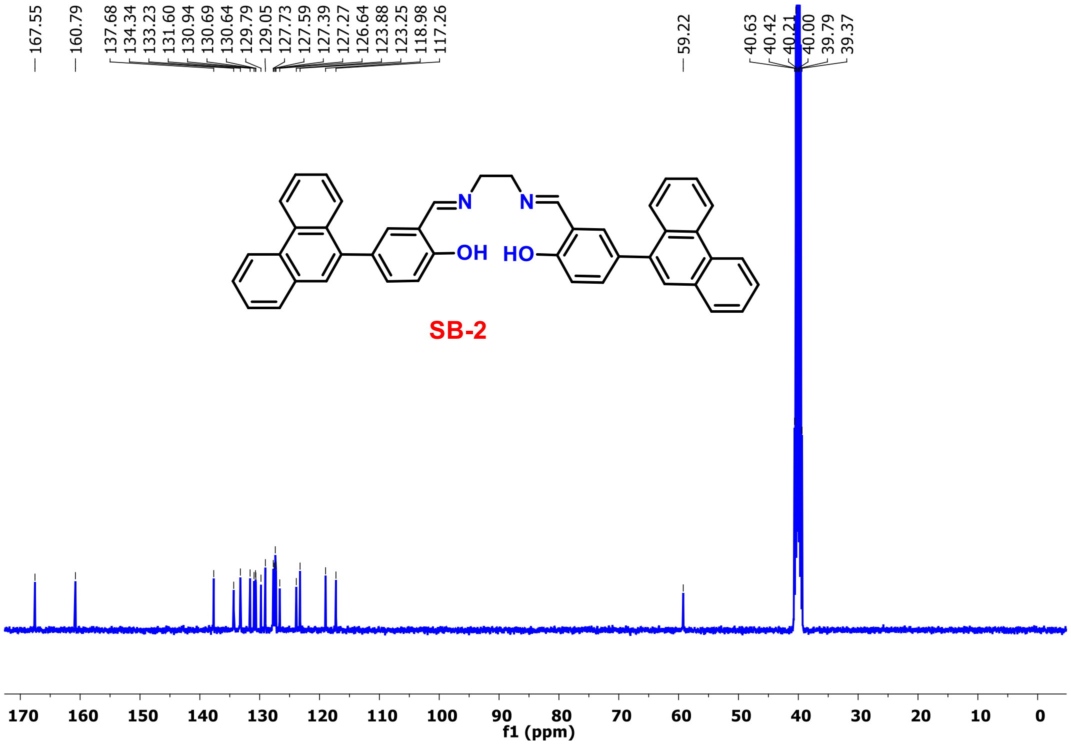

Figure S6. $^{13}C\{^{1}H\}$ NMR of **SB-2** (**DMSO-d6**, 126 MHz, RT).

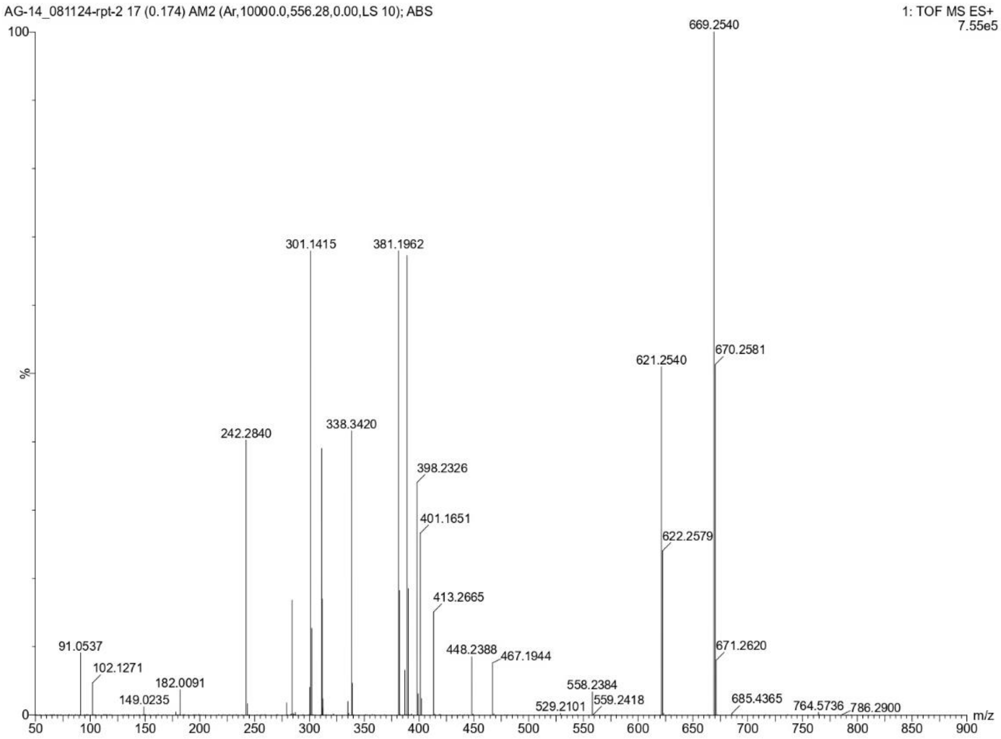

Figure S7. HRMS Spectrum of **SB-1**

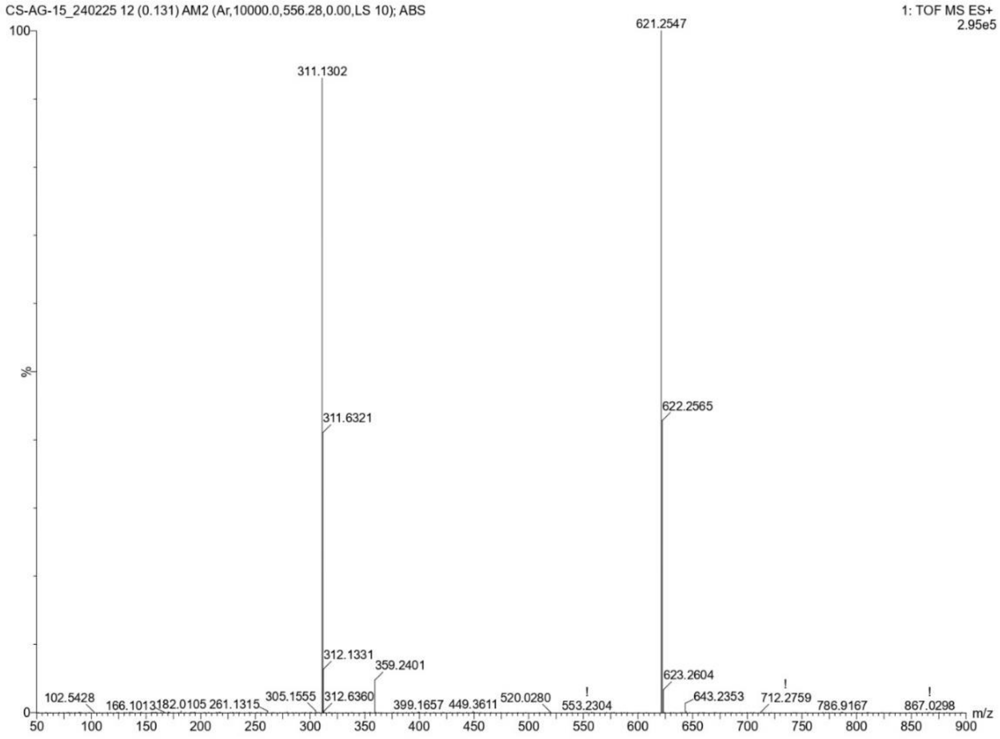

Figure S8. HRMS Spectrum of **SB-2**

2. Photophysical Properties

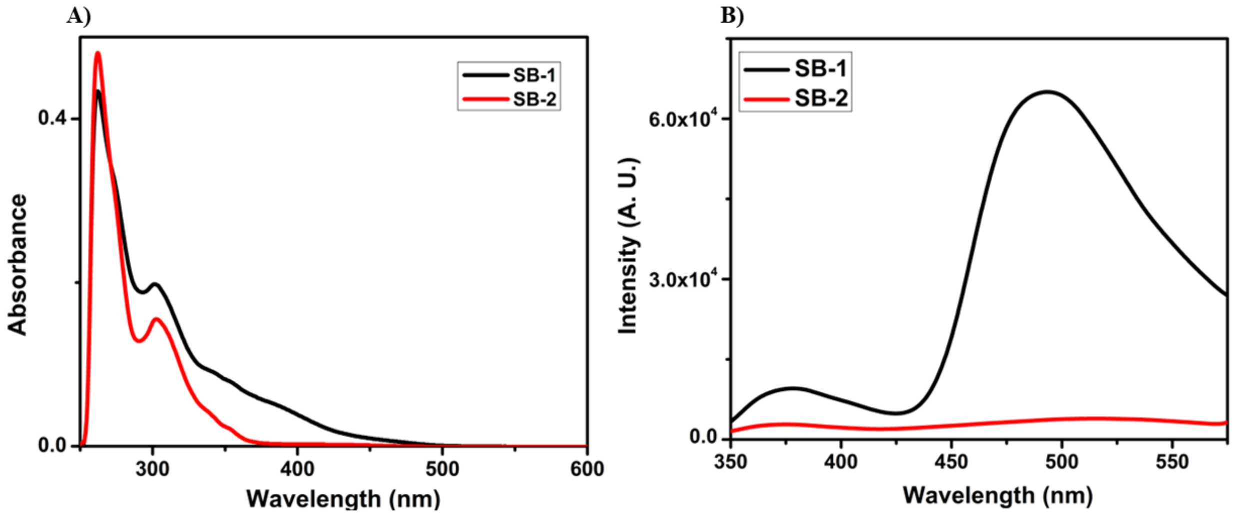

Figure S9: Absorbance (left) and Emission spectra (right) of **SB-1** and **SB-2** (10µM, in DMSO λ_{ex} = 302nm)

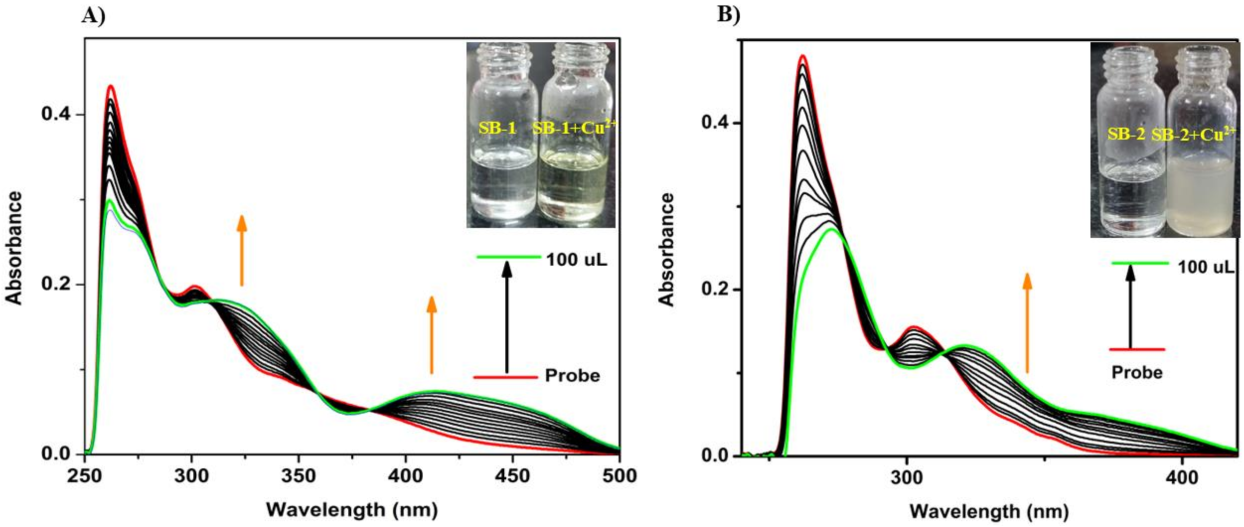

Figure S10. Absorbance spectral change of compounds (A) **SB-1** (left) and (B) **SB-2** (right) upon the addition of Cu^{2+} ions in DMSO solution (Inset photographs was taken in the presence of normal light).

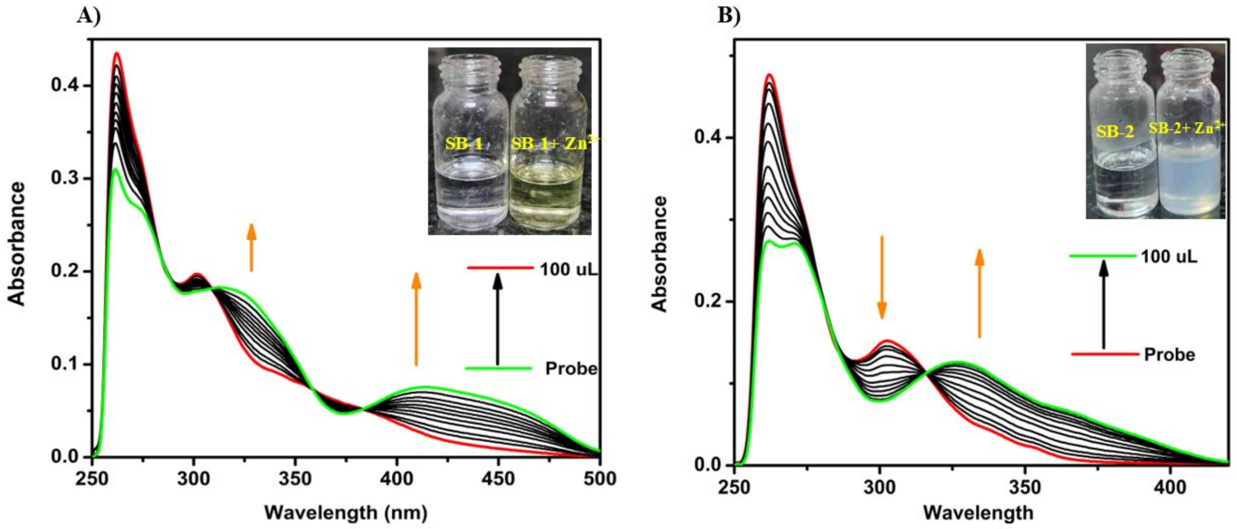

Figure S11: Absorbance spectral change of compounds (A) **SB-1** (left) and (B) **SB-2** (right) upon the addition of Zn^{2+} ions in DMSO solution (Inset photographs was taken in the presence of normal light).

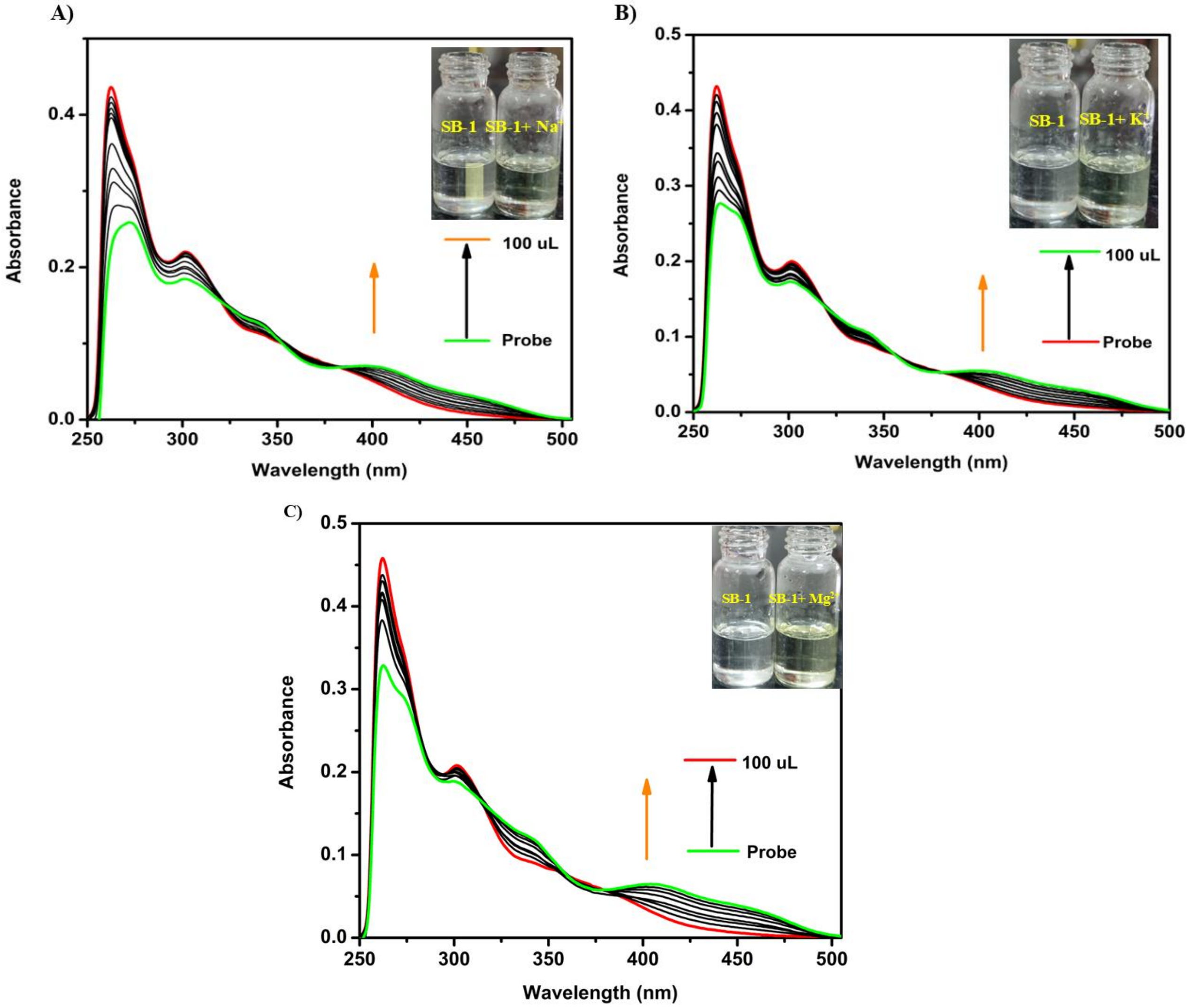

Figure S12. Absorbance spectral change of compound **SB-1** upon the addition of Na^{+}, K^{+}, Mg^{2+} ions in DMSO solution (Inset photographs was taken in the presence of normal light).

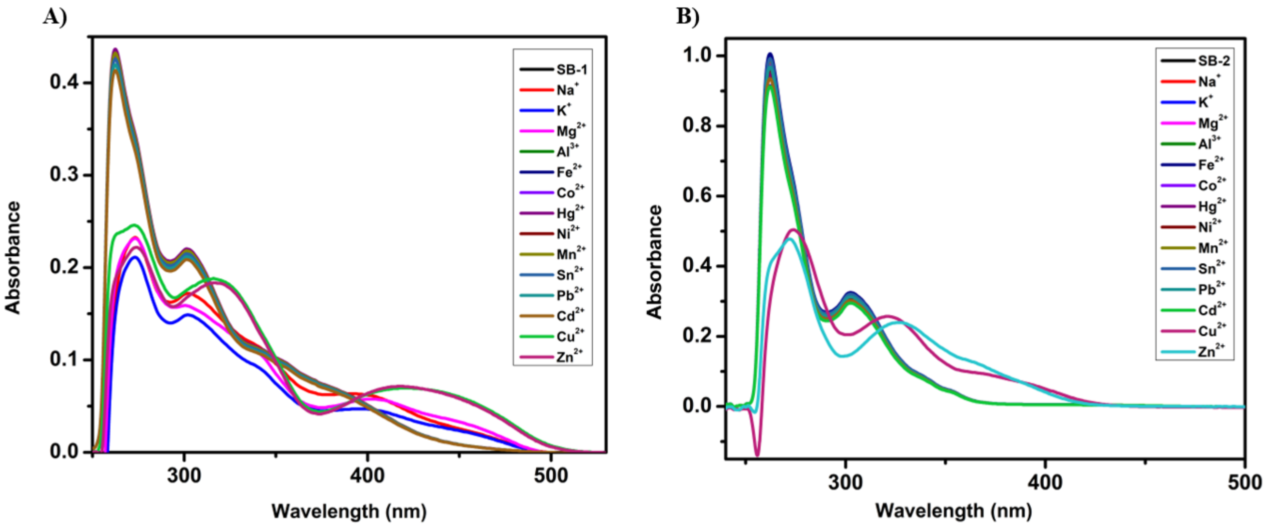

Figure S13. UV-Vis titration spectra of compounds (A) **SB-1** (left) and (B) **SB-2** (right) in DMSO solution with excess metal ions. Most metal ions caused no spectral changes, whereas Na^+, K^+, Mg^{2+}, Cu^{2+}, and Zn^{2+} induced absorption changes and a new band at 320 nm in **SB-1**, while Cu^{2+} and Zn^{2+} caused similar effects in **SB-2**.

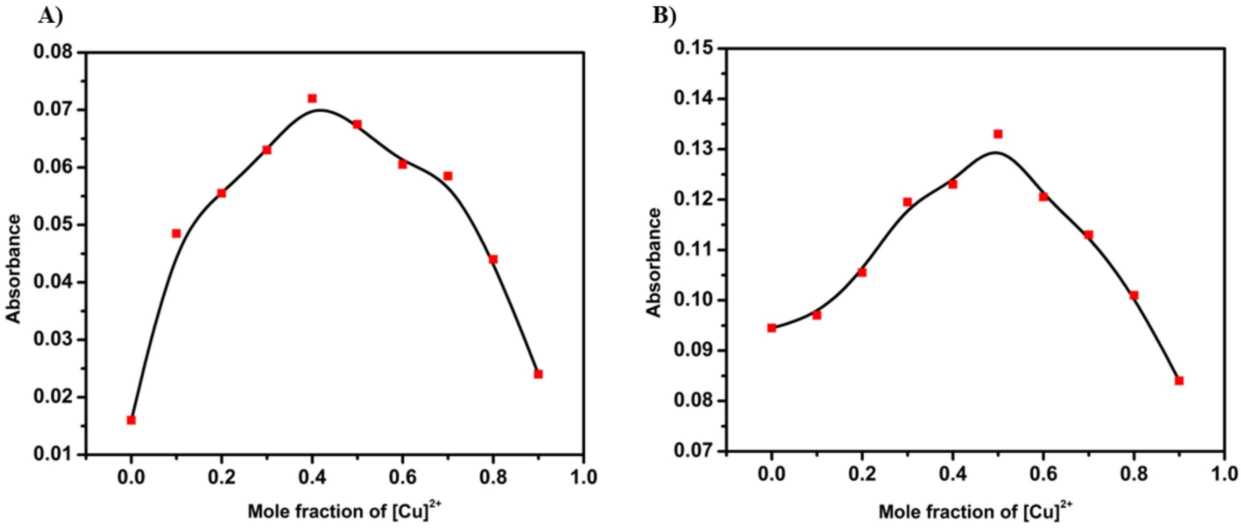

Figure S14. Job's plot according to the method of continuous variations, indicating the 1:1 stoichiometry for compounds (A) **SB-1** (left) and (B) **SB-2** (right) with Cu^{2+} ions in DMSO solution.

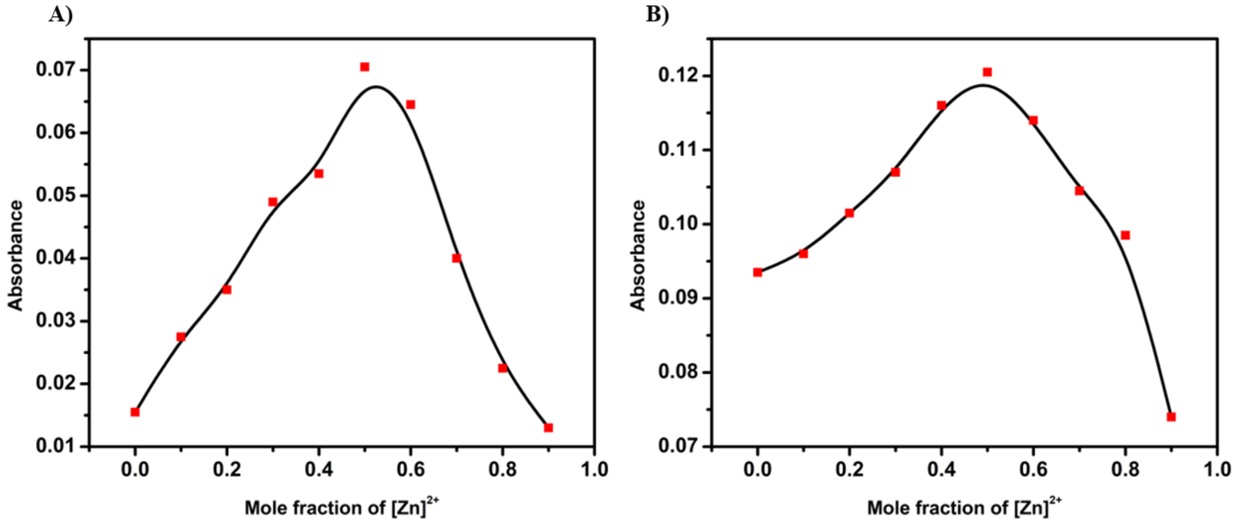

Figure S15. Job's plot according to the method of continuous variations, indicating the 1:1 stoichiometry for compounds (A) **SB-1** (left) and (B) **SB-2** (right) with Zn^{2+} ions in DMSO solution.

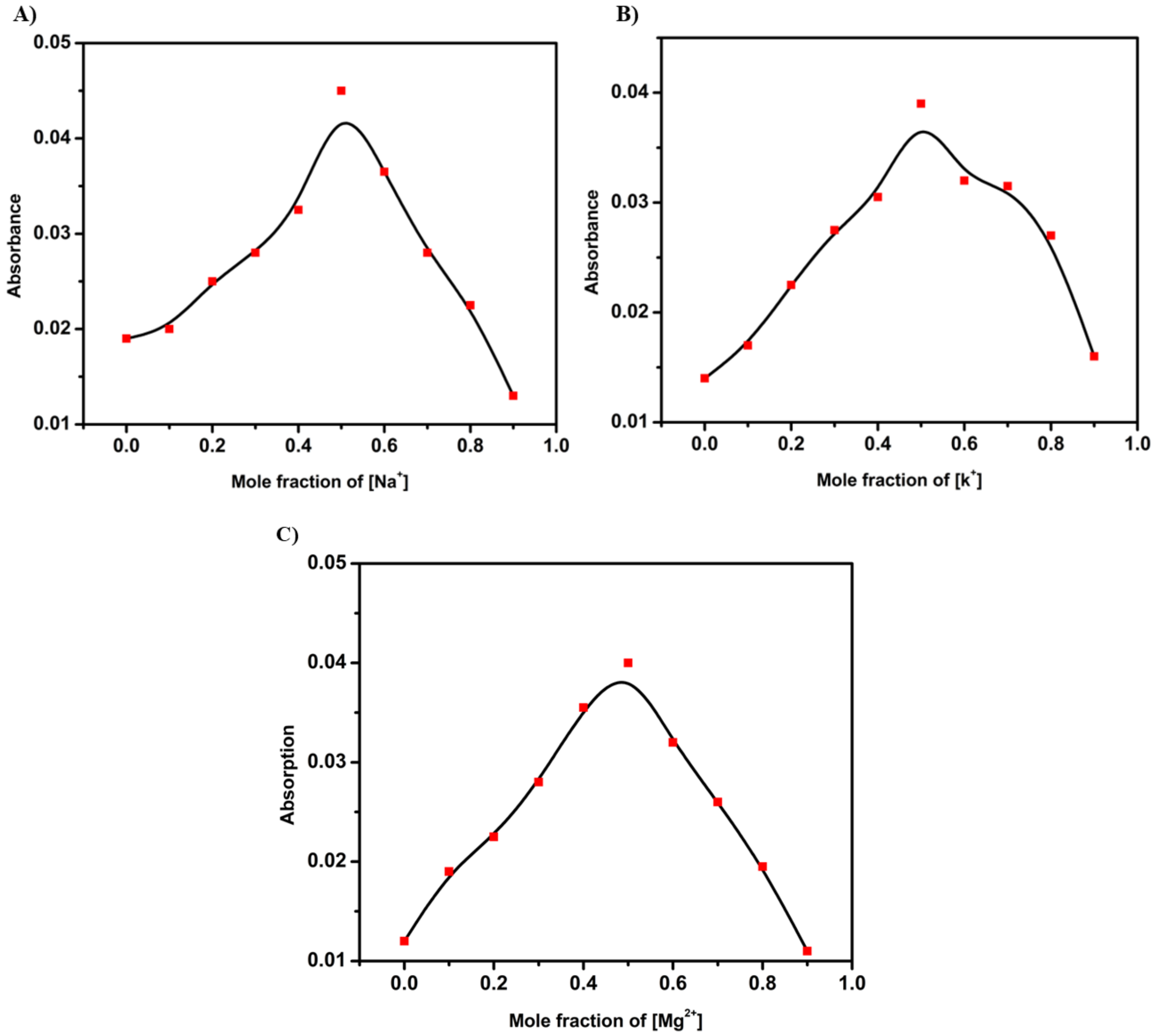

Figure S16: Job's plot according to the method of continuous variations, indicating the 1:1 stoichiometry for compound **SB-1** with (A) Na^{+}, (B) K^{+}, (C) Mg^{2+} ions in DMSO solution.

A) B)

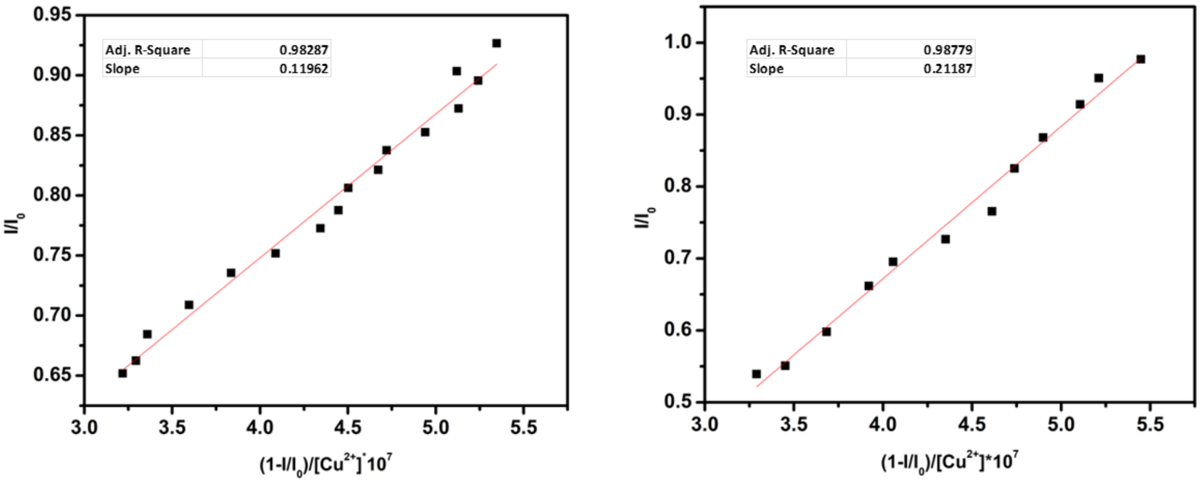

Figure S17: The (1 – I/I_0)/[Metal] vs I/I_0 plot for absorption titration data of Compounds (A) **SB-1** (left) and (B) **SB-2** (right) with Cu^{2+} ions in DMSO solution.

A) B)

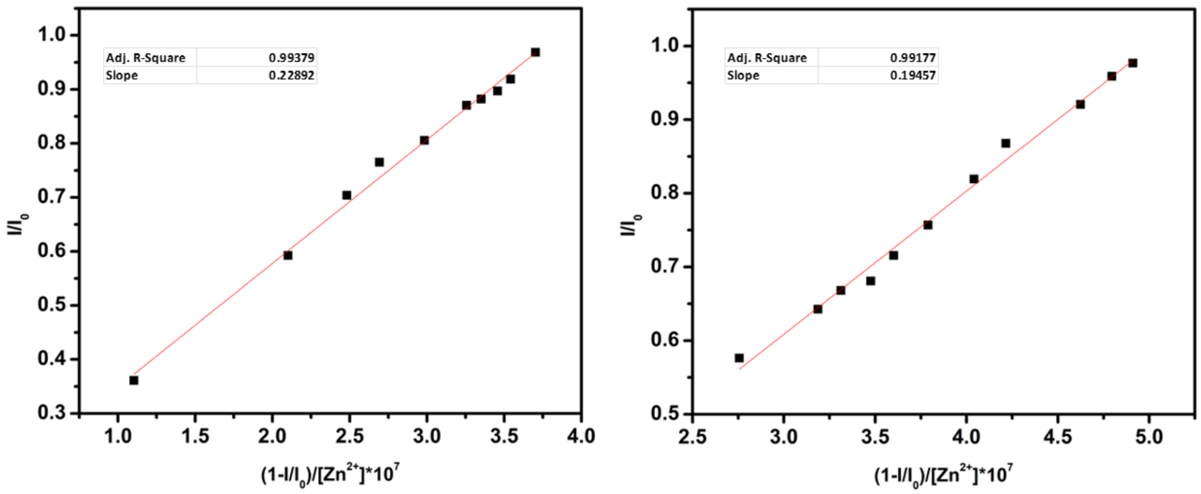

Figure S18: The (1 – I/I_0)/[Metal] vs I/I_0 plot for absorption titration data of compounds (A) **SB-1** (left) and (B) **SB-2** (right) with Zn^{2+} ions in DMSO solution.

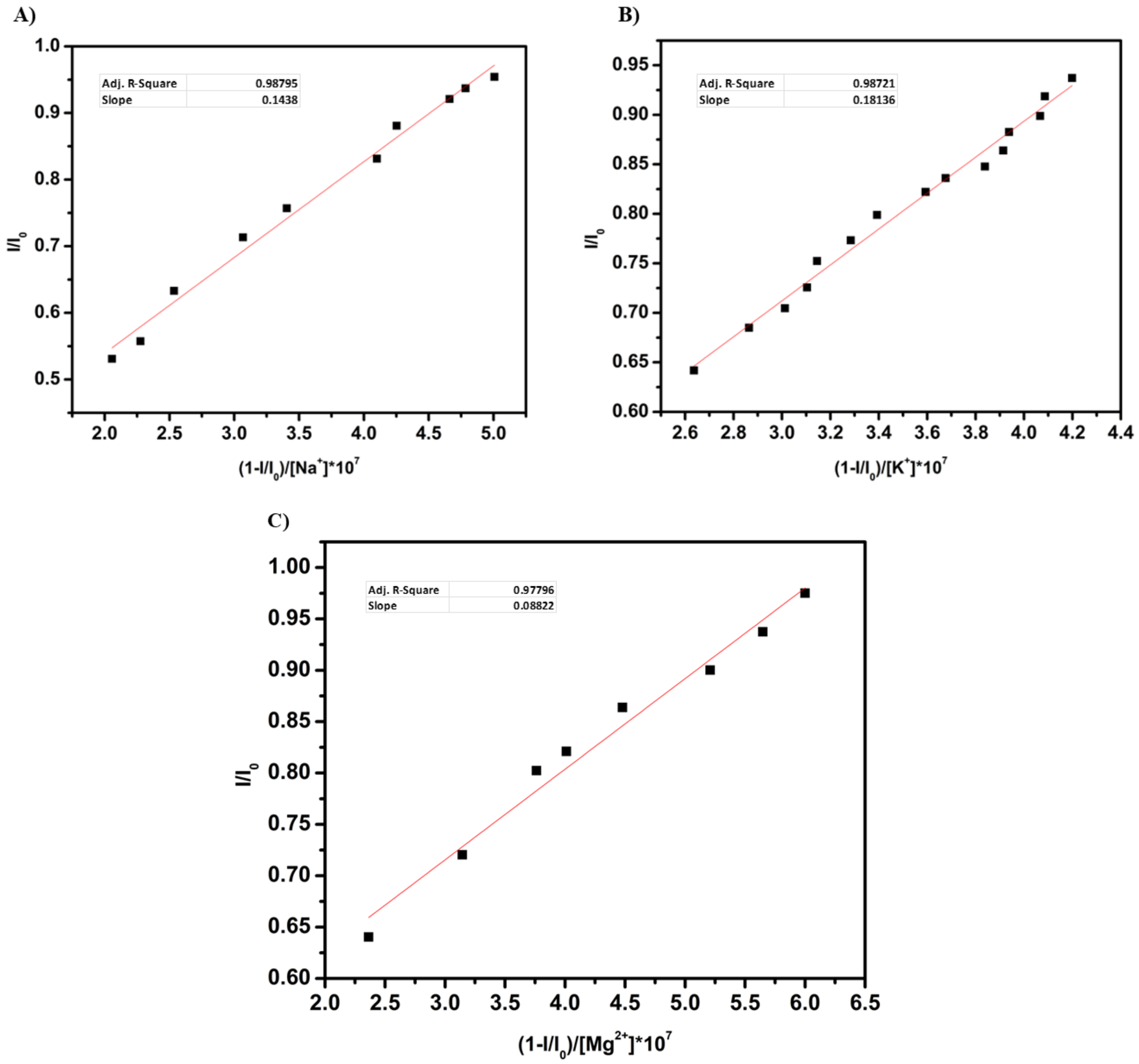

Figure S19. The (1 – I/I_0)/[Metal] vs I/I_0 plot for absorption titration data of compound **SB-1** with (A) Na^+, (B) K^+, (C) Mg^{2+} ions in DMSO solution.

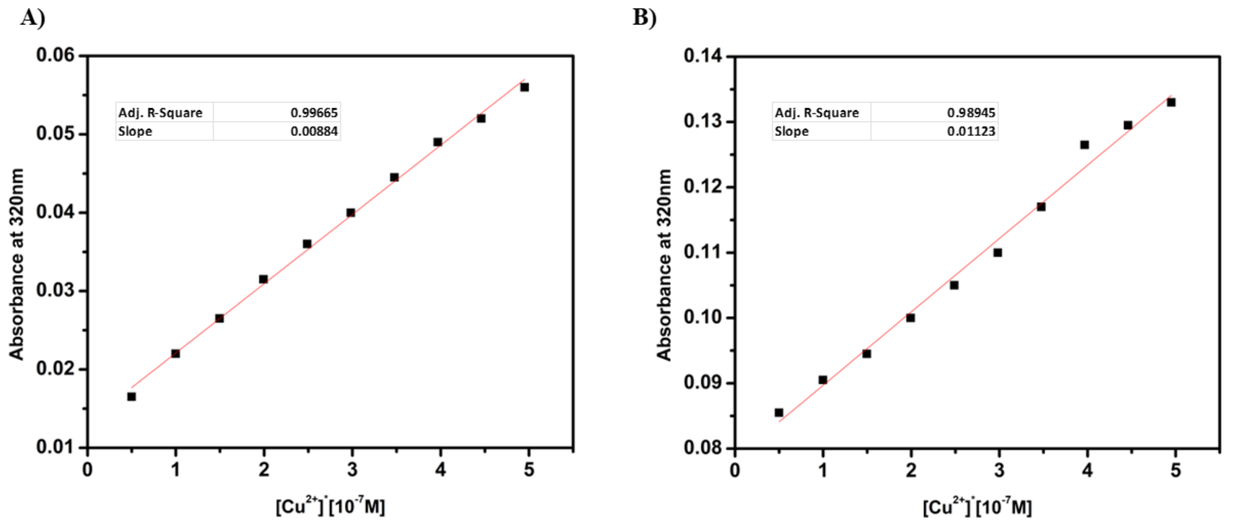

Figure S20. Absorbance at 320 nm vs [metal ions] plots for compounds (A) **SB-1** (left) and (B) **SB-2** (right) with Cu^{2+} ions in DMSO solution.

A)

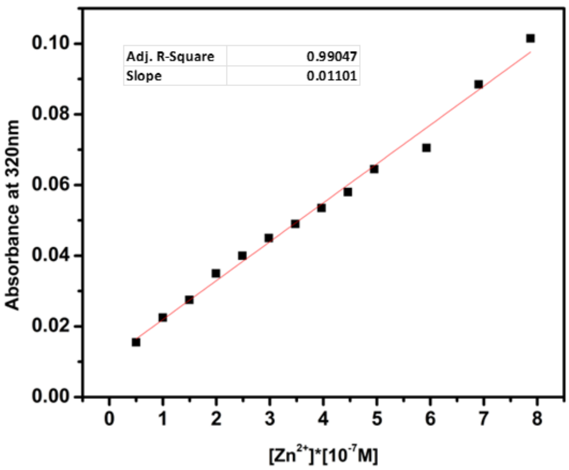

B)

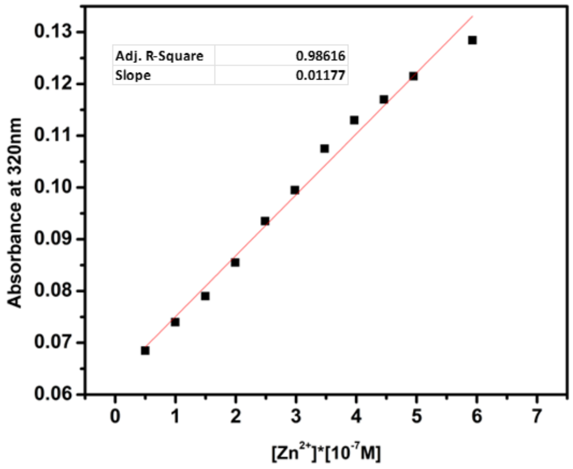

Figure S21. Absorbance at 320 nm vs [metal ions] plots for compounds (A) **SB-1** (left) and (B) **SB-2** (right) with Zn^{2+} ions in DMSO solution.

A)

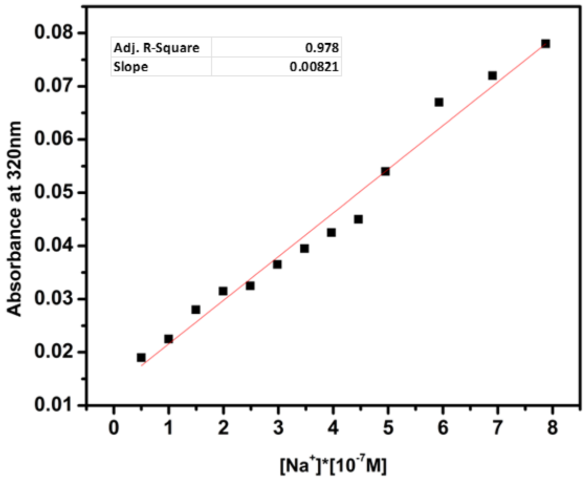

B)

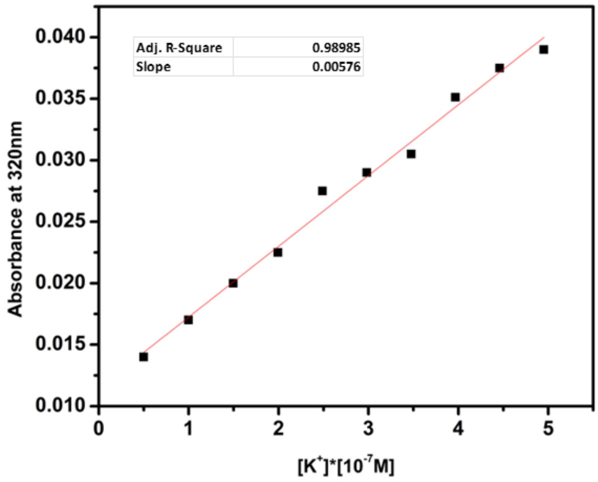

C)

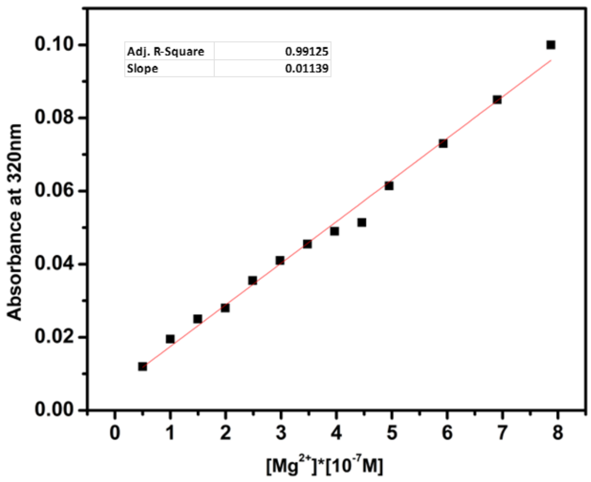

Figure S22. Absorbance at 320 nm vs [metal ions] plots for compound **SB-1** with (A) Na^+, (B) K^+, (C) Mg^{2+} ions in DMSO solution.

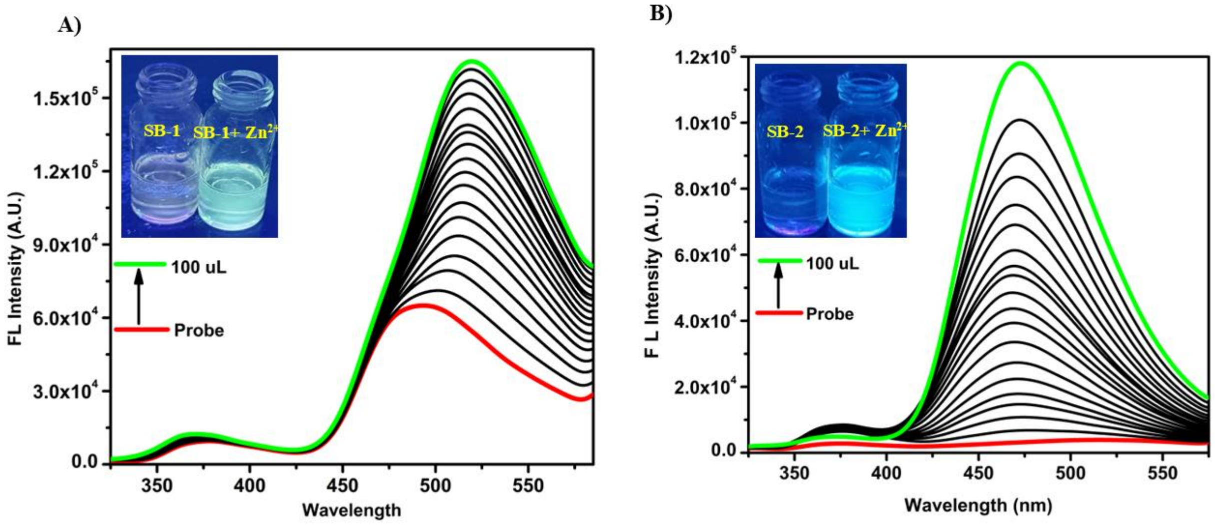

Figure S23. Emission spectral change of compounds (A) **SB-1** (left) and (B) **SB-2** (right) upon the addition of Zn^{2+} ions in DMSO solution. (Inset photographs was taken in the presence of fluorescence light).

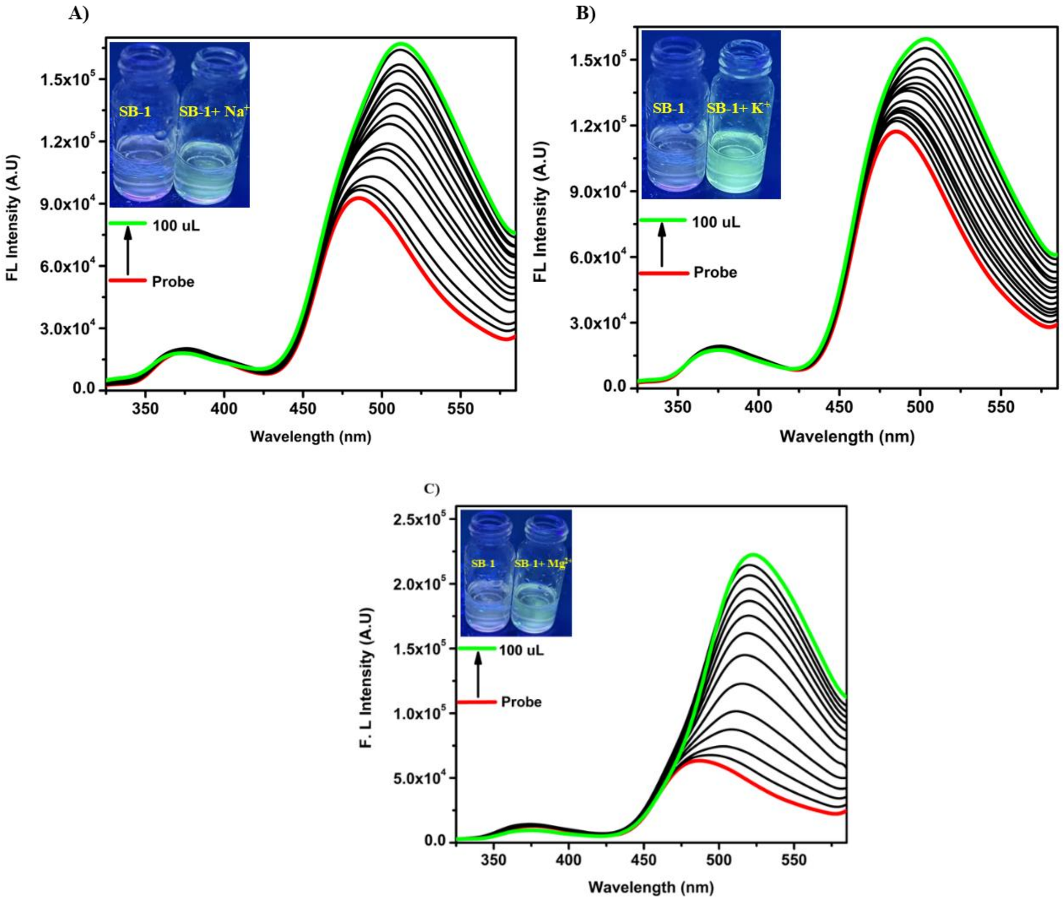

Figure S24: Emission spectral change of compound **SB-1** upon the addition of (A) Na^+, (B) K^+, (C) Mg^{2+} ions in DMSO solution. (Inset photographs was taken in the presence of fluorescence light).

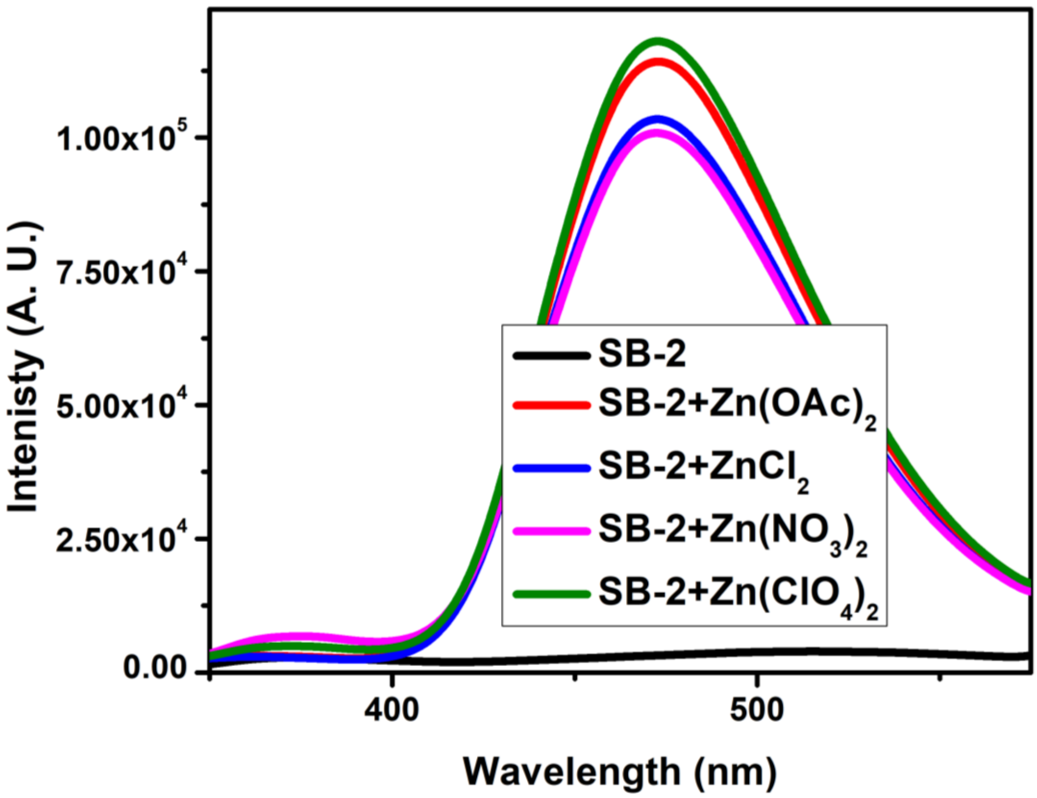

Figure S25. Fluorescence spectra of **SB-2** with different counter anions of Zn^{2+} ions

3. Detection limits Association constants and quantum yields of the synthesised probes

Table S1. Photophysical properties of compounds **SB-1** and **SB-2** with metal ions (Na^{+}, K^{+}, Mg^{2+}, Cu^{2+}, Zn^{2+}).

S. No	Compounds	Association constants ($\times 10^3$)	Detection limits ($\times 10^{-6}$ M)	Quantum yields[a]
1	**SB-1**			0.04
2	**SB-2**			0.01
3	**SB-1+Cu^{2+}**	1.19	0.05	NA
4	**SB-1+Zn^{2+}**	2.28	0.08	0.28
5	**SB-1+Na^{+}**	1.43	0.13	0.10
6	**SB-1+K^{+}**	1.81	0.10	0.12
7	**SB-1+Mg^{2+}**	0.88	0.08	0.32
8	**SB-2+Cu^{2+}**	2.11	0.10	NA
9	**SB-2+Zn^{2+}**	1.94	0.11	0.54

The PL quantum yields were calculated for the DMSO solution using the following equation: $\Phi = \Phi_R \times (I/I_R) \times (A_R/A) \times (\eta^2/\eta_R^2)$, here Φ is the quantum yield, I is the area under the PL band, A is the absorbance at λ_{ex}, and η is the refractive index of the solvent. Standard 0.1 M H_2SO_4 quinine sulfate ($\Phi_R = 0.577$) was used. The emission wavelength used was $\lambda_{ex} = 350$ nm.

4. ^{1}H NMR titration data

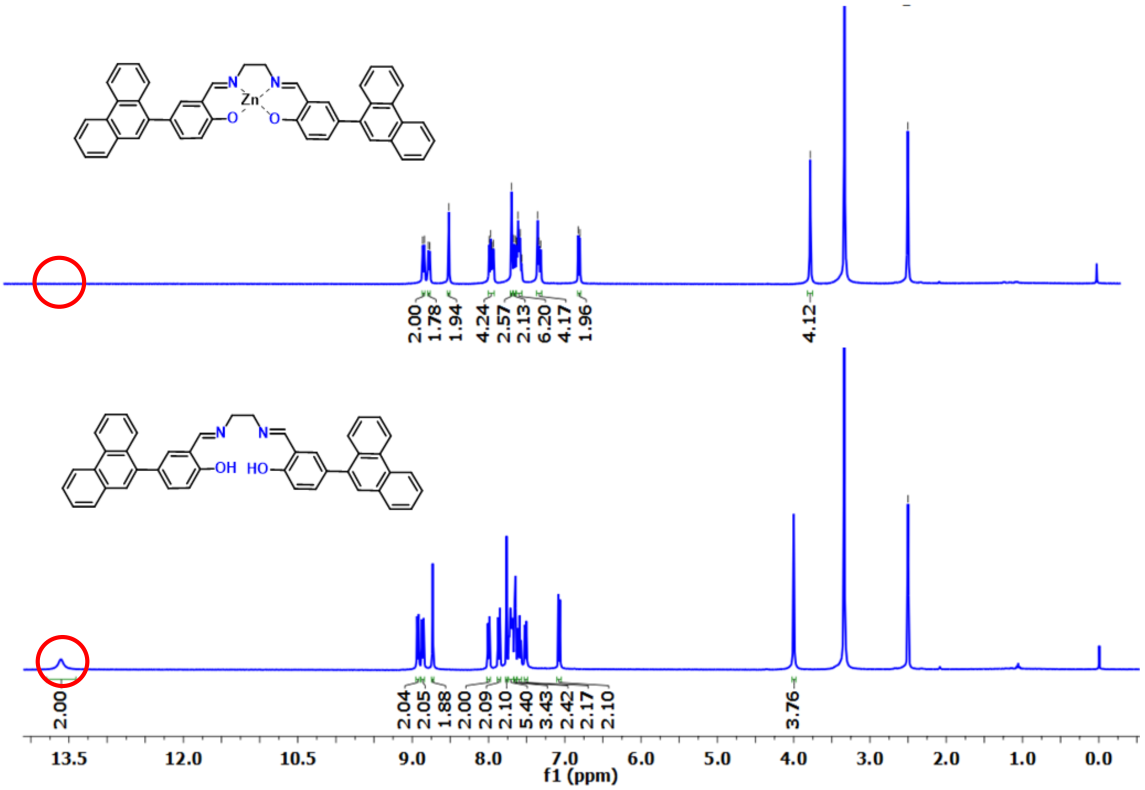

Figure S25. Compared ^{1}H NMR of **SB-2** and **SB-2**+Zn^{2+} (NMR titration in DMSO-d6)

5. DFT computational data

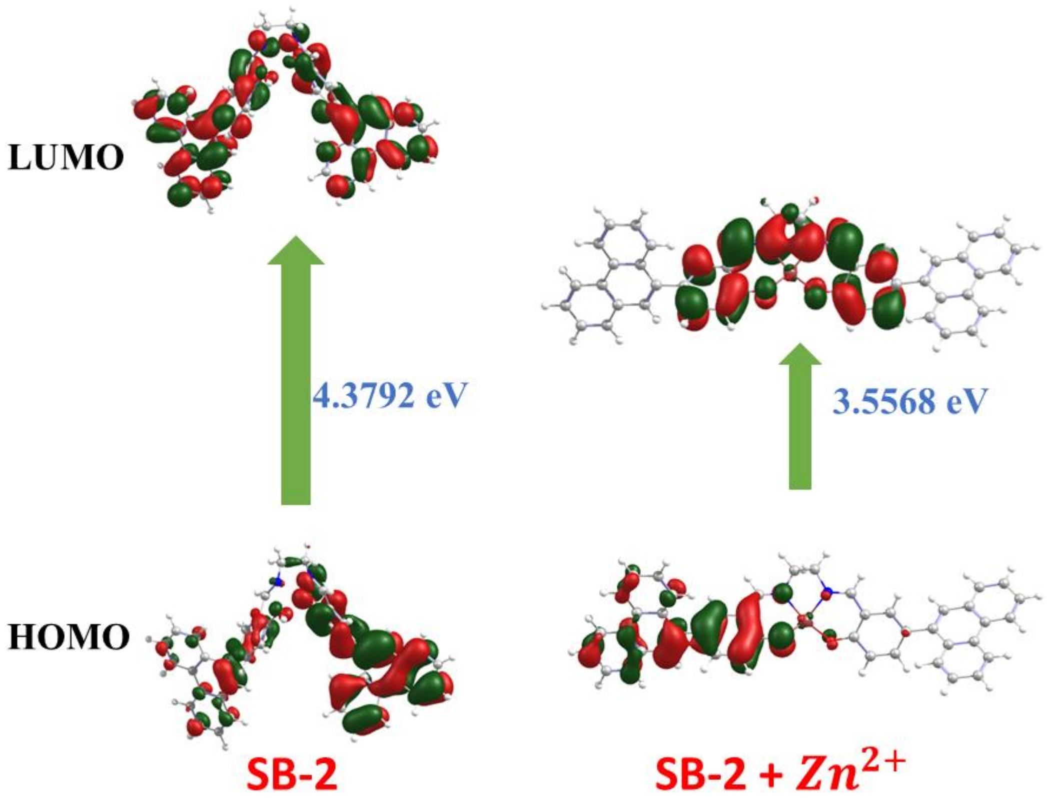

Figure.S26 Selected MOs of **SB-2** and **SB-2+Zn^{2+}** (not to scale; **isocontour value** = 0.02)

Table S2 Coordinates of **SB-1**:

6	0.556600204	5.542237691	-0.580389896
6	1.165643137	4.311845537	-0.835130214
6	0.584843626	3.109303627	-0.407217011
6	-0.659997480	3.151957012	0.274895611
6	-1.252007674	4.396418105	0.532431112
6	-0.652045332	5.585010030	0.111469529
1	1.026955258	6.459215661	-0.925015241
1	2.087580059	4.267172648	-1.408530600
1	-2.176530288	4.422107597	1.102812134
1	-1.131338984	6.536643659	0.324934815
7	1.136676345	1.860345511	-0.729592374
7	-1.208959433	1.957340388	0.765714521
6	2.387050433	1.665706239	-0.541244501

1	3.013369819	2.434087777	-0.058911994
6	-2.446072287	1.712841438	0.552650605
1	-3.055454635	2.385282923	-0.073201176
6	-3.185265566	0.553217830	1.064538252
6	-4.501804432	0.381467579	0.606937274
6	-2.671258913	-0.399189871	1.979072909
6	-5.318770743	-0.685551264	0.997977946
1	-4.888833913	1.107757115	-0.103577038
6	-3.477383007	-1.471176232	2.379483421
6	-4.774581312	-1.615055256	1.897289264
1	-3.079738954	-2.197080500	3.087279794
1	-5.380562453	-2.452056435	2.232702712
6	3.122204010	0.450064554	-0.905393941
6	4.479227603	0.388217589	-0.544264061
6	2.569648605	-0.658713050	-1.591252154
6	5.299177849	-0.710267144	-0.817794394
1	4.903792812	1.236435046	-0.012556611
6	3.380061670	-1.766032107	-1.873432821
6	4.717539471	-1.795107518	-1.494720503
1	2.951687913	-2.611582420	-2.409691166
1	5.322697873	-2.661998803	-1.744091625
8	1.267863926	-0.625544437	-1.967580762
1	1.058084639	-1.456454294	-2.424194263
8	-1.411598658	-0.246673909	2.456359270
1	-1.225182076	-0.974460055	3.071546576
6	9.708847161	1.460837411	-1.215652710
6	11.068698280	1.534269628	-0.989862595
6	8.937555340	0.401167421	-0.682333850
6	11.698512077	0.538484223	-0.219697967
6	9.568055427	-0.608029305	0.100020147

6	10.963372217	-0.506533649	0.310445864
6	7.532506980	0.321152065	-0.941367214
6	6.746221027	-0.689410400	-0.460511605
6	7.344262546	-1.713668680	0.379155859
6	8.749178664	-1.681931089	0.647366871
1	9.207884955	2.220160650	-1.811642477
1	11.650469504	2.353112471	-1.404110610
1	12.768977220	0.588648083	-0.039659193
1	11.480592653	-1.257120368	0.898056009
1	7.088404658	1.084148851	-1.576242772
6	6.559972438	-2.732898458	0.972527446
6	9.296577338	-2.700270479	1.462893963
6	8.510438972	-3.697121510	2.009198055
6	7.126131609	-3.708866265	1.769667656
1	5.488957374	-2.734266857	0.804170239
1	10.359411427	-2.703005145	1.677820398
1	8.963962078	-4.462940109	2.632666238
1	6.500394911	-4.477692153	2.214857638
6	-7.500418201	1.337496074	1.256360856
6	-8.476123838	2.311250777	1.347129973
6	-7.714212636	0.139143471	0.532214081
6	-9.715542259	2.117340373	0.714745327
6	-8.987844377	-0.074830366	-0.083717616
6	-9.961193217	0.946398833	0.022858302
6	-6.684270035	-0.881584166	0.433557613
6	-6.971624870	-2.058597195	-0.201477285
6	-8.238388419	-2.322207487	-0.811983509
6	-9.256582439	-1.327298659	-0.778671907
1	-6.551319966	1.482882205	1.759673344
1	-8.285821169	3.220107167	1.911458107

1	-10.486765817	2.880278418	0.777615527
1	-10.932345814	0.813262092	-0.440798834
1	-6.199785509	-2.820131433	-0.284423242
6	-8.478686485	-3.551722393	-1.469906133
6	-10.484198407	-1.615952053	-1.419894649
6	-10.698433912	-2.824947258	-2.057182435
6	-9.688850294	-3.805418592	-2.083597338
1	-7.686523807	-4.296751687	-1.485562334
1	-11.283139529	-0.882707435	-1.419620848
1	-11.652727873	-3.016400232	-2.540397793
1	-9.861916020	-4.753391621	-2.585598446

Table S3 Coordinates of **SB-1+Zn^{2+}**:

6	-2.615150313	1.380871240	-0.454998392
1	-3.273238585	2.240134276	-0.617733368
6	2.608384157	1.309083632	-0.648340975
1	3.266673294	2.119910598	-0.975466693
7	1.315095561	1.439264336	-0.794220363
7	-1.318442475	1.548767196	-0.495714979
6	3.271612068	0.206637789	-0.028764756
6	4.690604724	0.190415784	-0.099582415
6	2.585011277	-0.804195634	0.744548378
6	5.463754831	-0.771753227	0.526642330
1	5.177602059	0.960520039	-0.694284445
6	3.403882615	-1.765065697	1.403664799
1	2.895404203	-2.516382792	1.999768788
6	-3.285187936	0.133512230	-0.271786016
6	-2.618728064	-1.147558214	-0.360315837
6	-4.698348223	0.178837988	-0.130574573

6	-3.452829526	-2.301417320	-0.318987404
6	-5.484074126	-0.958041256	-0.057580356
1	-5.174125979	1.156821948	-0.096469033
1	-2.959580910	-3.265612294	-0.392583428
6	-0.703025305	2.730269278	-0.954024944
6	0.702390054	2.649691442	-1.176355256
6	-0.681755888	5.024493089	-1.754257279
6	1.371505719	3.753971286	-1.724060530
6	0.689089772	4.930902372	-2.011444756
1	2.434633888	3.696310689	-1.933845543
6	-1.368835792	3.932557088	-1.235699241
1	-2.434880775	4.020633862	-1.053776787
8	1.297753994	-0.872042846	0.897300148
8	-1.338309102	-1.308201043	-0.499075885
6	4.775797679	-1.757635520	1.285679868
1	5.359914009	-2.516440383	1.800681522
6	-4.817912949	-2.209659895	-0.163180320
1	-5.411265687	-3.119239877	-0.110112528
30	-0.008043487	0.024843126	-0.171117962
1	1.225967449	5.774786856	-2.434655875
1	-1.217212910	5.945137162	-1.966911352
6	-6.930463506	0.429110241	2.159661636
6	-7.573459690	1.118452629	3.169531983
6	-7.649058111	-0.174151015	1.098375438
6	-8.974035126	1.227513520	3.157188553
6	-9.077900449	-0.094349232	1.103833393
6	-9.703066006	0.626840091	2.148432034
6	-6.969544304	-0.897160118	0.035138223
6	-7.717237890	-1.542522909	-0.911974699
6	-9.147055396	-1.506092167	-0.931503287

6	-9.846402025	-0.764629750	0.063005871
1	-5.851262917	0.330213622	2.182696446
1	-6.996619711	1.566674628	3.974035334
1	-9.487483110	1.771108896	3.945728717
1	-10.784136548	0.708005879	2.172387730
1	-7.213298321	-2.080069478	-1.711879330
6	-9.870874356	-2.177873207	-1.945254673
6	-11.258803249	-0.727951972	-0.010358912
6	-11.946732773	-1.392242000	-1.009882185
6	-11.249582970	-2.126510253	-1.987864332
1	-9.317154924	-2.736952083	-2.695989033
1	-11.827134058	-0.171033402	0.726527781
1	-13.032014138	-1.345272169	-1.038538810
1	-11.794401694	-2.646853840	-2.770844494
6	7.292271463	1.479032218	1.264578825
6	8.109752309	2.551244445	1.565572119
6	7.807697989	0.289447982	0.693765857
6	9.488440921	2.470594546	1.307980832
6	9.217302194	0.191681912	0.466563581
6	10.023617188	1.312046379	0.777592756
6	6.944187347	-0.835747833	0.373079322
6	7.507989405	-1.995455348	-0.084961590
6	8.913824492	-2.143797193	-0.303314324
6	9.785490601	-1.046502568	-0.050510486
1	6.232277151	1.536028603	1.483949412
1	7.687285029	3.449452719	2.007964701
1	10.138054308	3.310999588	1.537419402
1	11.093562150	1.267259473	0.607104176
1	6.865496891	-2.837722301	-0.331056045
6	9.442149871	-3.362372105	-0.791881812

6	11.165334724	-1.225134577	-0.307014125
6	11.660970550	-2.425354632	-0.783902242
6	10.794264419	-3.507194233	-1.028806680
1	8.758724387	-4.187173436	-0.979947988
1	11.861237468	-0.412483962	-0.129615846
1	12.726309394	-2.531254324	-0.970126283
1	11.188268314	-4.448107673	-1.403055404

Table S4 Coordinates of **SB-2**:

6	-0.635954248	5.697520204	0.453391324
6	0.845588037	5.662712724	0.034373512
7	-0.948276243	4.570564152	1.311249512
7	1.076293723	4.604187571	-0.929957492
6	-1.771192062	3.705118246	0.866365907
1	-2.238539082	3.821838454	-0.126981620
6	1.848935651	3.652071920	-0.583026903
1	2.335721590	3.645928696	0.407882041
6	2.193692416	2.482694289	-1.408196197
6	3.060691785	1.535023050	-0.842500595
6	1.713421565	2.240749223	-2.718072039
6	3.467463098	0.370839429	-1.505640885
1	3.413457892	1.713804890	0.170268923
6	2.111354495	1.081419360	-3.393750781
6	2.970495148	0.161897553	-2.800538307
1	1.742662894	0.903864312	-4.403054935
1	3.269577509	-0.725164346	-3.351767409
6	-2.199451907	2.486838588	1.572620982
6	-3.097544504	1.640414377	0.902276949
6	-1.773468773	2.103584644	2.866267127
6	-3.582484165	0.445834445	1.444277512
1	-3.417449730	1.926764680	-0.096753598

6	-2.255904822	0.913501619	3.425896209
6	-3.141995007	0.097681698	2.731354948
1	-1.932842657	0.630884936	4.426865625
1	-3.508506969	-0.811014041	3.200129006
8	-0.904742767	2.895805140	3.545378060
1	-0.726897586	2.483958700	4.406614827
8	0.875395010	3.138475110	-3.297224210
1	0.650994090	2.815398525	-4.185071200
6	-7.963691292	0.119201700	-0.796441171
6	-8.967629435	-0.576436016	-1.439696372
6	-6.767919041	-0.525640000	-0.400515261
6	-8.801036764	-1.949049980	-1.703417400
6	-6.592048728	-1.913535127	-0.666503762
6	-7.640388600	-2.599134298	-1.324085420
6	-5.742868366	0.198840585	0.286369041
6	-4.575062238	-0.380652039	0.700551470
6	-4.338622329	-1.784540203	0.406758291
6	-5.346650393	-2.551102726	-0.259028280
1	-8.078736702	1.179101411	-0.581630986
1	-9.881142035	-0.070199745	-1.739404141
1	-9.587959794	-2.503631023	-2.207451501
1	-7.545198979	-3.657206966	-1.542263168
1	-5.925657406	1.246551576	0.512890859
6	-3.111857674	-2.411891675	0.734970614
6	-5.084900519	-3.917178387	-0.518116920
6	-3.887909488	-4.509671943	-0.162710253
6	-2.886535637	-3.747145895	0.461399695
1	-2.330211674	-1.822198388	1.200245540
1	-5.834688874	-4.522735837	-1.015098438
1	-3.720311771	-5.561609971	-0.377835299

1	-1.934955937	-4.202050291	0.722918136
6	6.204283145	0.964007555	-0.465334816
6	7.450563936	1.274400106	0.043532253
6	5.643069823	-0.327752605	-0.314976614
6	8.190663035	0.292472943	0.723102676
6	6.408583823	-1.337571323	0.349543916
6	7.678212496	-0.983398044	0.863500105
6	4.331086173	-0.651841700	-0.850007255
6	3.870537038	-1.936263650	-0.751841367
6	4.610730472	-2.979780457	-0.111567979
6	5.881272506	-2.691412353	0.463104755
1	5.643004099	1.719104791	-1.003953368
1	7.857880661	2.273088186	-0.088556167
1	9.170433282	0.529556320	1.128946807
1	8.276584592	-1.728213921	1.376385077
1	2.885523273	-2.178965805	-1.143480649
6	4.081566395	-4.289283869	-0.024778122
6	6.569145408	-3.745207793	1.109617893
6	6.033858225	-5.018753114	1.182356608
6	4.778543066	-5.297733248	0.609855215
1	3.108670789	-4.487479424	-0.468702286
1	7.538163039	-3.565035254	1.562109400
1	6.587480235	-5.806866322	1.685585846
1	4.362227826	-6.299645966	0.669309782
1	1.082294482	6.619421745	-0.450236145
1	-0.803269410	6.617735945	1.028867398
1	-1.261875724	5.737893623	-0.454543475
1	1.470377466	5.568529387	0.939048349

Table S5 Coordinates of **SB-2+Zn^{2+}**:

6	0.516735418	-2.984291286	-0.523493087

6	-0.589730467	-2.970523110	0.564349465
7	1.229431205	-1.717877073	-0.432976924
7	-1.276772408	-1.690413605	0.467522846
6	2.518050129	-1.675003026	-0.300483498
1	3.090851137	-2.604168181	-0.428063961
6	-2.563953478	-1.621661097	0.336370808
1	-3.155893096	-2.538175084	0.468010062
6	-3.301976015	-0.439306664	-0.011730096
6	-4.713437659	-0.511736714	0.085079167
6	-2.678573936	0.756461290	-0.528092922
6	-5.546834231	0.538110174	-0.269708231
1	-5.149968153	-1.427147092	0.479783213
6	-3.555544825	1.811493106	-0.905499745
6	-4.923657264	1.711414580	-0.767812592
1	-3.097394895	2.710758671	-1.305445390
1	-5.552022602	2.547340207	-1.065762831
6	3.280072459	-0.508102350	0.044980386
6	4.691022896	-0.610089530	-0.045525239
6	2.682233213	0.700703238	0.558809693
6	5.545228760	0.420409992	0.310353396
1	5.114186712	-1.536599908	-0.429285943
6	3.581724684	1.734980600	0.945184546
6	4.947501572	1.606403928	0.814062155
1	3.141410282	2.641057259	1.349818320
1	5.589640119	2.426078880	1.126687299
8	1.405192560	0.898503603	0.721757534
8	-1.397827413	0.922870210	-0.699660114
6	9.577047365	-2.098368517	1.628008049
6	10.941036809	-2.308874883	1.658297477
6	9.022195442	-1.010386036	0.913306453

6	11.794259292	-1.425859922	0.970205761
6	9.879868887	-0.114010696	0.214085755
6	11.272947469	-0.354752168	0.266753037
6	7.608656169	-0.788162590	0.906191538
6	7.023396503	0.252843333	0.238650942
6	7.862453864	1.164934384	-0.520949279
6	9.282681461	0.993159745	-0.521859546
1	8.904865680	-2.769041073	2.158451905
1	11.354842358	-3.148341440	2.210377301
1	12.869188944	-1.583805196	0.991047237
1	11.958759808	0.306535986	-0.251496234
1	6.985173237	-1.463123536	1.487716091
6	7.301568473	2.209895675	-1.295200434
6	10.065559354	1.907709563	-1.264902253
6	9.492252532	2.934408165	-1.991332554
6	8.095320482	3.082082119	-2.014893161
1	6.223023534	2.315669148	-1.329660266
1	11.145019187	1.804990105	-1.276718225
1	10.123770597	3.618468705	-2.551850998
1	7.638516847	3.874392300	-2.601668654
6	-7.291804835	-1.529783803	-1.541473138
6	-8.068931418	-2.523863798	-2.103978775
6	-7.845009059	-0.556830766	-0.673210972
6	-9.443625853	-2.579210843	-1.819432865
6	-9.251393803	-0.590783736	-0.411037009
6	-10.015744195	-1.627339513	-0.996860334
6	-7.023579440	0.486297731	-0.080306104
6	-7.625625930	1.455327077	0.675396141
6	-9.030437589	1.471005118	0.944980447
6	-9.859747346	0.438107848	0.422340373

1	-6.235297642	-1.476533390	-1.778447325
1	-7.618571574	-3.254182981	-2.770987430
1	-10.061507073	-3.360023637	-2.254766435
1	-11.082321155	-1.679667195	-0.808419194
1	-7.014398426	2.234197818	1.125388381
6	-9.598902968	2.489141557	1.746912415
6	-11.238975930	0.474332810	0.735549550
6	-11.774179344	1.479523464	1.520866754
6	-10.949371986	2.499101324	2.032462978
1	-8.947838947	3.267534752	2.138044110
1	-11.902781533	-0.295973400	0.358327909
1	-12.837856095	1.479792725	1.743245709
1	-11.374143040	3.286748818	2.648781654
1	-1.272731425	-3.821014392	0.431820708
1	1.182338002	-3.847710538	-0.386158708
1	0.040779587	-3.065522131	-1.509661542
1	-0.115377050	-3.055778687	1.550926297
30	-0.007214991	-0.167771594	0.012962448
